# Network-Guided Analysis of Genes with Altered Somatic Copy Number and Gene Expression Reveals Pathways Commonly Perturbed in Metastatic Melanoma

**DOI:** 10.1371/journal.pone.0018369

**Published:** 2011-04-08

**Authors:** Armand Valsesia, Donata Rimoldi, Danielle Martinet, Mark Ibberson, Paola Benaglio, Manfredo Quadroni, Patrice Waridel, Muriel Gaillard, Mireille Pidoux, Blandine Rapin, Carlo Rivolta, Ioannis Xenarios, Andrew J. G. Simpson, Stylianos E. Antonarakis, Jacques S. Beckmann, C. Victor Jongeneel, Christian Iseli, Brian J. Stevenson

**Affiliations:** 1 Ludwig Institute for Cancer Research, Lausanne, Switzerland; 2 Swiss Institute of Bioinformatics, Lausanne, Switzerland; 3 Department of Medical Genetics, University of Lausanne, Lausanne, Switzerland; 4 Service of Medical Genetics, Centre Hospitalier Universitaire Vaudois, Lausanne, Switzerland; 5 Protein Analysis Facility, Center for Integrative Genomics, Lausanne, Switzerland; 6 Ludwig Institute for Cancer Research, New York, New York, United States of America; 7 Department of Genetic Medicine and Development, University of Geneva, Geneva, Switzerland; 8 Institute for Genomic Biology and National Center for Supercomputing Applications, University of Illinois at Urbana-Champaign, Champaign, Illinois, United States of America; University of Texas M. D. Anderson Cancer Center, United States of America

## Abstract

Cancer genomes frequently contain somatic copy number alterations (SCNA) that can significantly perturb the expression level of affected genes and thus disrupt pathways controlling normal growth. In melanoma, many studies have focussed on the copy number and gene expression levels of the *BRAF*, *PTEN* and *MITF* genes, but little has been done to identify new genes using these parameters at the genome-wide scale. Using karyotyping, SNP and CGH arrays, and RNA-seq, we have identified SCNA affecting gene expression (‘SCNA-genes’) in seven human metastatic melanoma cell lines. We showed that the combination of these techniques is useful to identify candidate genes potentially involved in tumorigenesis. Since few of these alterations were recurrent across our samples, we used a protein network-guided approach to determine whether any pathways were enriched in SCNA-genes in one or more samples. From this unbiased genome-wide analysis, we identified 28 significantly enriched pathway modules. Comparison with two large, independent melanoma SCNA datasets showed less than 10% overlap at the individual gene level, but network-guided analysis revealed 66% shared pathways, including all but three of the pathways identified in our data. Frequently altered pathways included WNT, cadherin signalling, angiogenesis and melanogenesis. Additionally, our results emphasize the potential of the *EPHA3* and *FRS2* gene products, involved in angiogenesis and migration, as possible therapeutic targets in melanoma. Our study demonstrates the utility of network-guided approaches, for both large and small datasets, to identify pathways recurrently perturbed in cancer.

## Introduction

Somatic copy number alterations (SCNA) are a recurrent characteristic of malignant cancers [1], [2], [3]. The amplification and subsequent over-expression or, conversely, deletion and loss of expression of key regulators of cell proliferation, senescence or death have been shown in many cases to contribute significantly to the progression from the normal to the malignant state [4], [5], [6], [7]. Therefore, the discovery and characterization of chromosomal regions involved in SCNA and of the genes encoded in them has been a crucial contributor to our understanding of the molecular mechanisms of carcinogenesis.

The methods used to detect and characterize SCNA have evolved significantly over the last decades. Initial cytogenetic observations have been supplemented with Southern blots and quantitative PCR. Almost twenty years ago, the availability of BAC clones delineating a tiling path through the entire human genome made it possible to detect SCNA in a genome-wide fashion, but with limited resolution [8]. More recently, oligonucleotide-based arrays have enabled comparative genome hybridizations (CGH) at high resolution, and CGH has become the method of choice to detect copy-number variations [9], [10], [11]. A recent SNP-based survey [1] of 3,131 copy-number profiles derived from over 26 different types of cancer has provided a dramatic illustration of the power of high-throughput techniques in distinguishing random alterations in the genome from those that may have a direct impact on tumorigenesis.

Genomic alterations in many tumors, especially at late stages in their development, are so extensive that the copy-number status of individual genes or chromosomal regions can vary over a very wide range of values. A mixture of chromosomal rearrangements and focal expansions can create genomic landscapes that are very difficult to analyze using standard CGH techniques. Moreover, the exact boundaries of SCNA or the expression status of the genes encoded within them are usually not known, precluding a thorough assessment of their impact on the phenotype of the cancer cells. It has recently been proposed that SNP arrays may be better suited for the determination of copy number states in tumor samples because the analysis of data derived from such arrays can make use of allelic imbalance information in addition to hybridization intensity [12].

In the present study, we analyzed the genome-wide copy-number status of seven highly aneuploid metastatic melanoma cell lines and determined the expression of their genes using a sequencing-based approach. We show that a combination of SNP-based and CGH arrays is necessary to obtain a reliable estimate of the true copy-number status of the entire genome in the face of extensive genomic instability, and that the combination of copy-number and expression status provides powerful clues as to the possible role of genes encoded within SCNA in tumorigenesis. Moreover, we show that a protein-based network-guided analysis of SCNA-affected genes with altered expression in our data and two published datasets [13], [14] identifies pathways commonly altered in melanoma.

## Results

### CGH and SNP arrays are required to comprehensively document somatic copy-number alterations in metastatic melanoma cell lines

**Figure 1:**
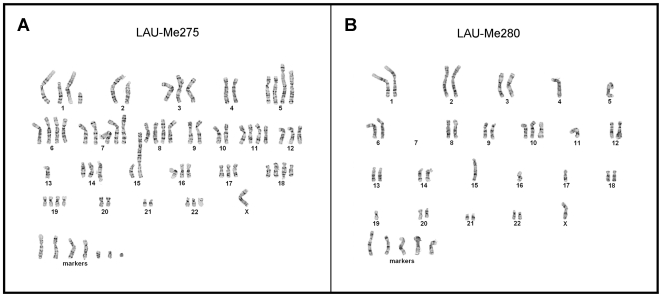
Karyotypes of two malignant melanomas. Representative karyotype (Giemsa stain) for LAU-Me275, one of the most hyperploid melanoma (here 76 chromosomes including 7 markers); and LAU-Me280, the most extensively deleted line (42 chromosomes including 5 markers).

We analyzed seven low-passage melanoma cell lines that were established from metastases (see Table 1) together with matched controls from the same patients (see Materials and Methods). Karyotyping of the melanoma cell lines revealed extreme levels of aneuploidy. For example, LAU-Me280, the most extensively deleted line, had a per cell content of 34 to 42 chromosomes (median: 40), whereas LAU-Me275, one of the most amplified melanomas, harbored 68 to 81 chromosomes (median 73.5) (see Fig. 1). Additionally, the presence of many unassigned chromosomal fragments (markers) made it difficult to determine the true level of aneuploidy.

**Table 1:** Melanoma cell lines.

Melanoma	Site	BRAF mutation	Number of chromosomes (karyotype)
**LAU-Me280.R.LN**	Lymph node	G593M, L597R	34–42
**LAU-Me246.M1**	Skin	V600E	45–82
**LAU-T618A**	Skin	wt but NRAS mutation (Q61R)	55–71
**LAU-T50B**	Skin	V600E	65–71
**LAU-T149D**	Visceral	V600E	68–81
**LAU-Me275**	Lymph node	V600E	68–81
**LAU-Me235**	Skin	K601E	73–103

In initial CGH (Agilent 244k) experiments we observed that in LAU-Me275, and other highly hyperploid cell lines, the hybridization ratios between cancer cells and matched controls did not reflect the chromosome-wide aberrations observed in the karyotypes. For example tetraploid regions were measured as triploid or less by the CGH arrays (see Fig. S1). We considered whether this was due to the normalization protocol and subsequent segmentation analysis. Using technical replicates of LAU-Me275 DNA, we tested three independent normalization schemes, two of which were specifically developed for cancer genome analysis (see Materials and Methods) and found that the methodology proposed by Chen and colleagues [15] was the most reproducible (Spearman correlation 0.96; see Fig. S2). We then partitioned the genome into regions reflecting copy number changes and assigned copy number using two independent classification methods (see Materials and Methods). Since neither of these classification methods gave entirely satisfactory results (see Fig. S4 and Methods S1), we developed a Gaussian Mixture Model (GMM) approach that was highly reproducible based on a technical replicate analysis (Spearman correlation 0.9).

The GMM method found only 42 regions in the LAU-Me275 genome that were amplified (CN≥4; see Table 2). This number was less than expected based on the karyotype analysis, which documented a high number of arm-level chromosome amplifications (see Fig. 1). Thus, while CGH-based methods are well adapted to document differences in copy number status between the genomes of normal cells derived from different individuals, our results clearly show that they are inadequate to deal with the large-scale rearrangements and amplifications typical of hyperploid cancer cells. The most likely reason is that the total DNA content of cancer cells is too different from that of normal cells to allow a robust experimental normalization. Given this limitation, we asked whether SNP arrays might be better suited to detect chromosome-wide changes in a highly amplified genome.

**Table 2:** Number of genes affected by SCNA in seven melanoma cell lines.

CGH arrays	LAU-Me280	LAU-Me246	LAU-T618A	LAU-T50B	LAU-T149D	LAU-Me275	LAU-Me235	Unique gene count
Deletion	3668	4281	986	3656	108	122	1059	10711
Arm-level amplification	222	0	549	99	998	42	0	1884
Focal amplification	0	0	0	26	379	0	4	409
SNP arrays	LAU-Me280	LAU-Me246	LAU-T618A	LAU-T50B	LAU-T149D	LAU-Me275	LAU-Me235	Unique gene count
Deletion	2294	3157	2	113	70	2	39	5544
Arm-level amplification	0	0	16584	1033	3477	16398	10384	19496
Focal amplification	213	0	978	438	894	1853	161	4055

Number of genes affected by somatic deletions, arm-level amplifications (≥4 copies but <1 copy above the chromosome arm baseline) and focal amplifications (≥4 copies and ≥1 copy above the chromosome arm baseline), as measured using SNP or CGH arrays.

**Figure 2:**
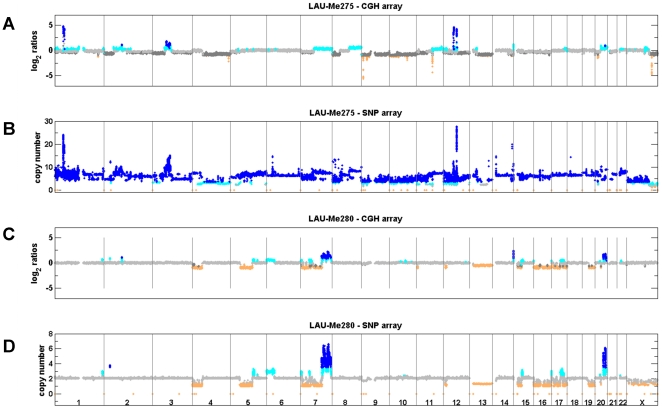
Copy number analysis using CGH and SNP arrays. **A.** and **B.** shows the analysis of LAU-Me275 on CGH and SNP arrays. **C.** and **D.** shows results for LAU-Me280. Probe/SNP are plotted as a function of their genomic position on the X axis. Y axis for CGH arrays corresponds to hybridization ratios. Y axis for SNP arrays corresponds to the predicted copy number. Colors indicate a copy number state (orange<2 copies; gray = 2 copies; cyan = 3 copies; dark blue>3 copies). Dark gray in the CGH panels indicates regions identified as diploid in the analysis, but where the karyotype analysis indicated copy neutral or deleted states, possibly due to cell heterogeneity.

**Figure 3:**
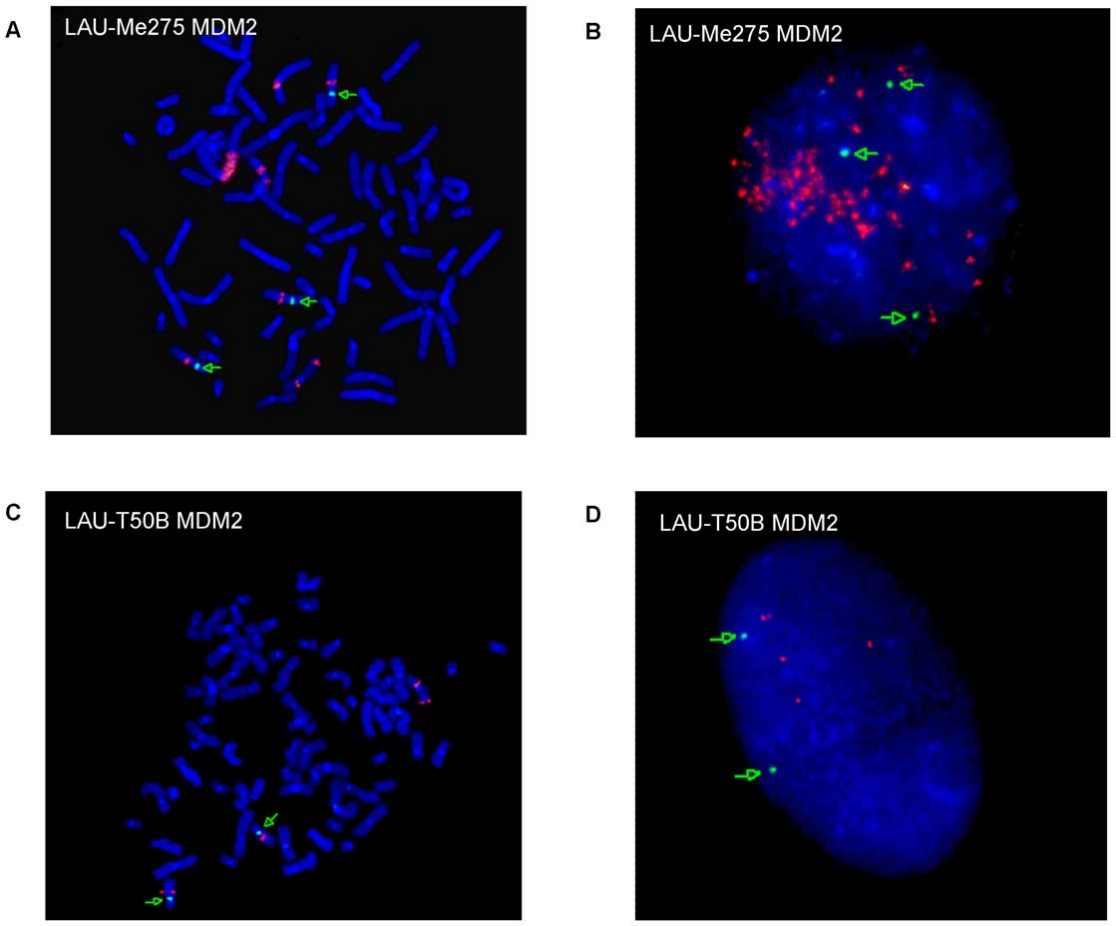
Determination of MDM2 copy number by FISH. The *MDM2* gene was assayed in two melanoma samples (LAU-Me275 and LAU-T50B) derived from the same patient. Panels **A** and **C** show a metaphase and **B** and **D** an interphase. *MDM2* probe is in red; centromere-specific probe is in green. FISH shows amplification for both LAU-Me275 (more than eight copies) and LAU-T50B (four copies). Metaphase-FISH helps to identify homogeneously staining regions and Interphase-FISH to estimate the copy number.

We hybridized DNA from LAU-Me275 to Illumina 1M SNP arrays and analyzed the signals using the OverUnder algorithm [16], which uses minor allele frequencies in heterozygous loci to improve copy number estimation. These results correlated well (Spearman correlation 0.77) with a technical replicate analyzed on the Affymetrix SNP platform (see Fig. S5C and S5D), and indicated that 18,251 genes in the LAU-Me275 genome had a copy number of at least four (see Table 2). Within this group, 132 genes had undergone focal amplifications of at least 10-fold. These SNP-based results were more consistent with the karyotype observations. For example, CGH had predicted two copies of chromosome 7p (Chr7p) and three copies of Chr7q (see Fig. 2), while the SNP results indicated three and five copies, respectively (see Fig. 2 and 3), which was more consistent with the cytogenetic data (see Fig. 1).

Therefore, based on our findings with LAU-Me275, we determined the SCNA in the six other melanoma cell lines using both CGH (Agilent 244k) and SNP (Illumina 1M) array platforms. Identification of genes within amplifications or deletions was determined as described in Materials and Methods, and the number of SCNA for each cell line is given in Table 2. In all cases CGH predicted more deletions than did SNP arrays, agreeing with our initial observations using LAU-Me275. Also, with the exception of the LAU-Me280 cell line, amplifications were better predicted by SNP arrays. This bias is evident in a graphical representation of the intersection between CGH and SNP predictions (see Fig. S7) and with CGH/SNP genome-wide copy number profiles (see Fig. S8). These results confirmed our conclusion that CGH is more suitable for detecting deletions while SNP arrays are better for identifying amplifications.

### Few SCNA-genes are recurrent in different melanoma cell lines

A potential problem with SCNA studies performed in isolation is that they cannot assess the expression status of the genes contained within the altered genomic regions. Amplified genes are not necessarily highly expressed, and the exact boundaries of deletions may or may not encompass a gene of interest. We reasoned that the combination of precise copy number determination and gene expression measurement would allow us to highlight with much higher confidence those genes whose expression is affected by SCNA (SCNA-genes) in the melanoma cell lines. We therefore analyzed gene expression in each of the melanoma cell lines by RNA-seq using the Roche/454 pyrosequencing method. Additionally, we performed RNA-seq on a pool of epidermal melanocytes to determine a reference level of expression for each gene in normal melanocytic cells (see Materials and Methods).

We first looked for genes within focal amplifications with at least two-fold over-expression relative to the reference melanocytes (see Table S1). Only *KIAA0090*, a protein coding gene of unknown function not previously associated with cancer, was affected in three melanomas (see Table S2). A further 56 genes were altered in two melanomas, but the only known cancer-related gene was *MDM2*, an oncogene previously demonstrated to be amplified in sarcoma, glioma, colorectal and other cancers including melanoma [5], [17]. In LAU-Me275, *MDM2* was 3.9-fold over-expressed relative to melanocytes and had a copy number greater than ten (as predicted by SNP array). By contrast, in LAU-T50B, *MDM2* was predicted by SNP array to be diploid (CN = 2), and by CGH to be duplicated (CN = 3). This potential difference between these cell lines is intriguing because they were derived from metastases surgically removed from the same patient at a 12 year interval. We therefore determined the copy number status of the *MDM2* gene in these two samples using fluorescent in situ hybridization (FISH). In LAU-Me275, the fluorescence signal indicated that at least 8 *MDM2* copies were present at the locus on Chr12 in addition to a homogeneously staining region on Chr5 (see Fig. 3A and 3B) which is in agreement with results from the SNP arrays. In LAU-T50B, FISH revealed a total of four *MDM2* copies, two on Chr12 and two located on an unidentified chromosome (see Fig. 3C and 3D), which is higher than the copy number estimated by CGH and SNP arrays. Re-investigation of the raw SNP data for LAU-T50B showed that there was indeed a small amplification signal at *MDM2*, but this had not been detected using our optimization parameters (see Methods S1). This highlights the challenge of determining optimal parameters that are usable on a genome-wide scale for all samples in a study.

We next derived a list of genes within deletions detected by CGH that were expressed in melanocytes but not in the melanoma cell lines (see Table S1). We reasoned that such genes are likely to be enriched for melanocyte functions that have been lost during tumorigenesis. The vast majority of such genes (554) were private to a single melanoma sample; seventy genes were shared by two samples; and only ten genes were shared by three melanomas: *ADAMTSL1*, *ARMC4*, *DLL1*, *HSD17B3*, *LOC441177*, *OSTCL*, *PARK2*, *PLXDC2*, *SLC24A2* and *ULBP3* (see Table S2).

**Figure 4:**
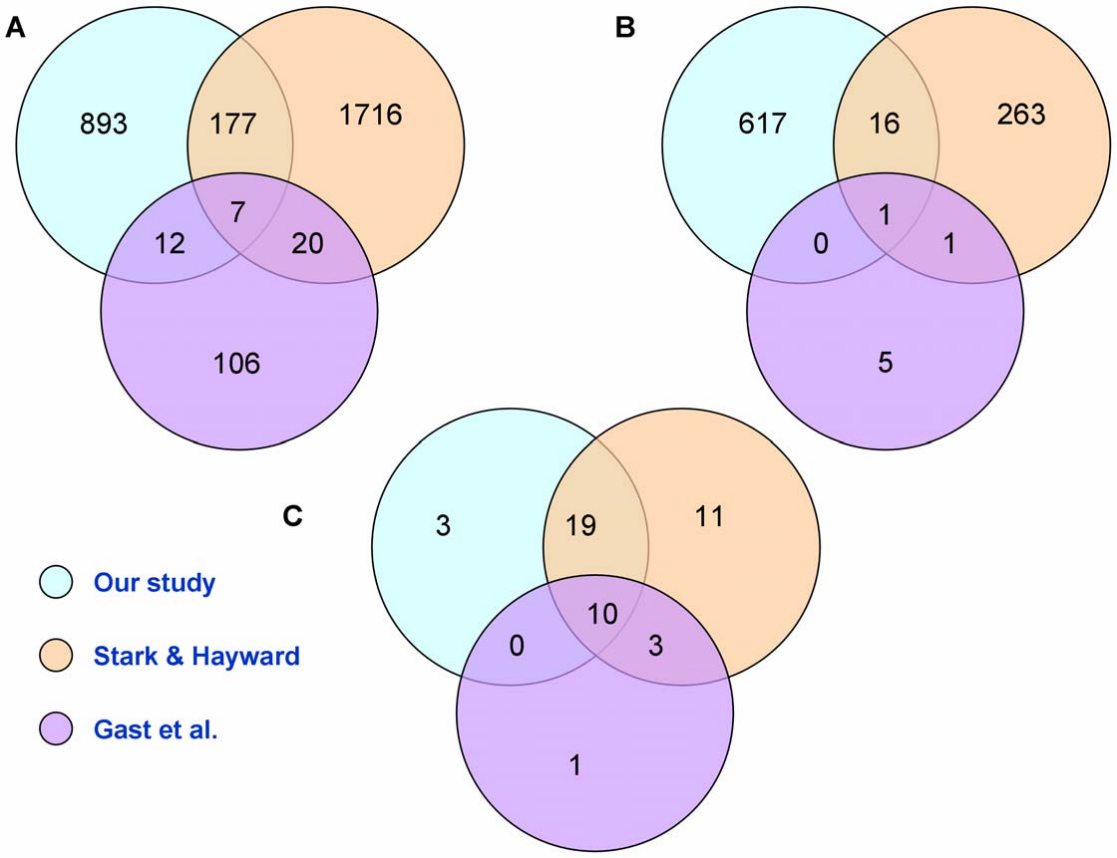
Intersection between our dataset and two published datasets of SCNA-genes and derived pathways. **A.** Intersection between amplified genes in published melanoma datasets (Stark and Hayward 2007; Gast et al., 2010) and our list of over-expressed genes within focal amplifications **B.** Intersection between genes within homozygous deletions from the Stark and Hayward and Gast et al. datasets and our list of non-expressed genes within deletions **C.** Intersection between pathways found significantly affected by SCNAs from our analysis of the three datasets.

Altogether, we identified a total of 1,710 SCNA-genes affected by amplification or deletion (summarized in Table S2; complete dataset in Table S3). To determine the relevance to melanoma of this set of altered genes from our small sample set, we compared it to gene lists in two published studies that used larger melanoma collections [13], [14]. These two studies provided a list of genes recurrently affected by amplification or deletion in 76 (primary and metastatic) and 60 (metastatic) melanoma samples, respectively. Only 196 of our 1,089 amplified genes (p-value<0.001, see Fig. 4A), and 17 of our 634 genes within deletions (p-value<0.007, see Fig. 4B) were present in the Stark and Hayward or Gast et al. datasets. Surprisingly, the number of genes common to the two published gene sets was also small (27 amplified genes and 2 genes within deletions; p-values<0.005), and demonstrates the difficulty to identify commonly affected genes relevant to tumor progression even within larger melanoma collections.

### Correlation between mRNA and protein levels

A potential problem with assessing gene expression by mRNA quantitation is that this may not reflect cellular protein levels. To check whether SCNA-genes were also expressed at the protein level in the melanoma cell lines, we performed a SILAC proteomic analysis (stable isotope labeling with amino acids in cell culture [18], [19]). This technique is used to quantify relative protein amounts as measured by mass spectrometry in extracts from cells grown in unlabeled (light) medium and cells grown in (heavy) medium containing non-radioactively-labeled amino acids. Since the normal human melanocytes did not grow well in the labeling media, heavy isotopes were incorporated into the Lau-Me275 cell line, and protein quantification in all cases was relative to this sample. After stringent data filtering (see Materials and Methods), we investigated whether transcript levels reflected protein levels as detected by SILAC. As shown in Fig. S11 there was a moderate but significant correlation between global mRNA and protein levels in all samples (mean Spearman correlation 0.62).

### Pathways significantly enriched in SCNA-genes are recurrent in melanoma

As described above, we found that very few of the SCNA-genes were altered in more than one melanoma cell line, which is not unexpected given the small number of samples in our dataset. An idea popular in the current literature is that signaling pathways, rather than individual genes, are recurrently perturbed in cancer [20]. To determine whether the SCNA-genes from different melanoma cell lines shared membership of one or more cellular pathways, we investigated whether the proteins encoded by the SCNA-genes were connected in known human protein interaction networks (see Materials and Methods). Out of a total of 1,563 proteins analyzed, 377 (24%) were connected within the network, and clustering of the proteins based on the topology of the sub-networks identified 14 protein networks, or ‘clusters’, containing at least five significantly connected members. For each cluster, we identified genes belonging to known signaling and metabolic pathways including nine clusters that significantly overlapped known pathways (FDR≤0.05; listed in Table S4). Following detailed manual annotation, the resulting pathways were ranked according to the number of contributing melanomas and to the number of SCNA-genes involved in the pathway. The pathways common to at least four melanoma samples are shown in Table 3 (for the complete list see Table S4). Interestingly, the vast majority of the recurrent pathways we identified involve signal transduction and have been implicated in one or more cancer types. In addition, that we identified ten pathways common to at least five of the melanoma samples confirms the idea that protein network-guided analysis is a good method for detecting recurrently affected pathways in small datasets.

**Table 3:** Pathways identified by network-guided analysis.

Pathway	#Melanomas	Genes	#genes
G protein signaling	6	ADORA1, ADRA2A, CHRM1, CHRM5, DRD2, GNAO1, GNB3, GNG4, HTR1F, OPRL1, PLCB2, RGS10, RGS11, RGS14, RGS19	15
WNT signaling (includes Apoptosis and Hedgehog signaling)	6	CDH19, CDH2, CDH4, DVL1, FRAT1, FZD8, PCDH17, PCDH9, SFRP1, WNT11, WNT16, WNT2B, WNT4, WNT5B	14
Cadherin signaling	6	ACTG2, CDH19, CDH2, CDH4, FZD8, PCDH17, PCDH9, WNT11, WNT16, WNT2B, WNT4, WNT5B	12
Melanogenesis	6	CAMK2A, CAMK2G, DVL1, FZD8, NRAS, WNT11, WNT16, WNT2B, WNT4, WNT5B	10
Angiogenesis	5	BRAF, DVL1, EFNB2, EPHA3, EPHB2, FGF1, FRS2, NRAS, PIK3R3, PRKCZ, SFRP1, WNT2B, WNT5B	13
Axon guidance (migration and adhesion)	5	CDK5, EFNB2, EPHA3, EPHB2, EPHB6, FES, NRAS	7
MAPK signaling	5	DUSP1, DUSP12, DUSP2, FGF1, FGF14, FGFR4, MAPK9	7
TGF beta signaling	5	ACVRL1, AMHR2, FOXH1, LEFTY1, SMAD9, TGFB1, TLL2	7
Alzheimer disease	5	CHRM1, CHRM5, PKN3, PRKCZ	4
FGF signaling	5	FGF1, FGF14, FGFR4, FRS2	4
Calcium signalling	4	CAMK2A, CAMK2G, CHRM1, CHRM5, GNAO1, GRIN2C, PRKCZ, RGS10, RGS11, RGS14, RGS19	11
Huntington_disease (vesicle-mediated transport)	4	ACTG2, CLTB, GRIN2B, GRIN2C, GRIN3A, KALRN	6
Neuroreceptor (Muscarinic, Metabotropic)	4	GRIN2B, GRIN2C, GRIN3A, KCNQ2, PKN3, PRKCZ	6
Cell cycle (G1 progression)	4	CCNA1, CDC20, CDC26, CDKN2B, HDAC1	5

In our search for genes recurrently affected by SCNA, we found only ∼10% overlap between our list of SCNA-genes and those derived from studies with much larger sample sizes [13], [14]. To determine if this was also true at the level of pathways, we performed a protein network-guided analysis as described above on each of these datasets using the published gene lists (neither study originally presented this type of analysis). Detailed annotation and comparison of the results for each dataset is given in Table S4. In contrast to what we had found at the gene level, all but three of our pathway modules were also present among those identified from one or both of the published gene datasets (see Fig. 4C). Ten pathway modules (angiogenesis, EGF, ERBB, integrin signaling, long term potentiation, MAPK, natural killer cell mediated toxicity, PDGF, regulation of actin cytoskeleton and VEGF) were common to all three datasets, and the combined overlap with our pathway dataset was 66%. Thus, the majority of pathways defined by SCNA affected genes in our melanoma samples were recurrent in the three datasets, whereas the individual genes were not.

An additional benefit of the protein network-guided approach is that it generates a list of genes affected by SCNA that contributed significantly to a given pathway (see Table S4). Although two-thirds of the pathways were common between our dataset and the published datasets, only two genes, *NRAS* and *BRAF*, were present in all three (see Table S5). Of the genes shared by two datasets, four were components of the angiogenesis pathway, including *EPHA3* and *FRS2*. We noted also that several members of the WNT (*WNT3A*, *4*, *5B*, *7A*, *9A*, *11*, *16*) or cadherin (*CDH2*, *4*, *9*, *12*, *17*, *18*, *19*) gene families were affected by SCNA in only one dataset, further reinforcing the idea that different genes can potentially alter the same pathway (WNT or cadherin) in different melanoma samples.

## Discussion

Our goal to identify somatic copy number aberrations in metastatic melanoma cell lines revealed extreme levels of aneuploidy characteristic of this cancer type [21], [22], and complicated the application of standard CGH array protocols [9], [10], [11]. Nevertheless, using our GMM method we were able to demonstrate that although CGH arrays fail to identify all large-scale amplifications, they are able to detect deletions very efficiently, including genes having lost expression compared to melanocytes (see Table 2 and Fig. S7). Conversely, SNP arrays, which measure hybridization intensities for both alleles at heterozygous loci, allow the consideration of an additional parameter (the so-called B-allele frequency) and greatly improve the measurement of DNA copies beyond the normal diploid complement (as implemented in the OverUnder algorithm, Attiyeh et al., 2009; see Fig. S6). We did notice, however, that this algorithm systematically detected deletions located in sub-telomeric regions for both tumors and controls, which indicates a systematic bias and suggests that the algorithm is optimized to detect duplications and amplifications but not deletions. Therefore, it can be argued that CGH and SNP techniques should be combined to obtain a reliable assessment of all copy number states from deletion to high-level focal amplification.

To enrich for genes that might be involved in the oncogenic process, we focused on two groups: focally amplified genes that were over-expressed relative to melanocytes; and deleted genes with no expression in the melanoma cell lines, but that were expressed in normal melanocytes. In the first group, *MDM2* [5], [17] was the only cancer gene amplified and over-expressed in more than one melanoma sample. Comparison of genes amplified in our samples with published gene lists from two large melanoma studies (Stark and Hayward 2007; Gast et al., 2010) while revealing very little overlap (see Fig. 4) did identify *BRAF*, *MDM2*, and *NRAS*, genes known to be important in melanoma [5], [17], [23], [24], [25], [26], [27], [28], [29]. In the second group, ten genes were deleted in three of the melanoma samples (see Table S2). These genes are located on Chr6q25, Chr6q27, Chr9 or Chr10p, consistent with previous observations that both arms of chromosomes 9 and 10 and Chr6q frequently undergo hemizygous deletion or copy neutral LOH in melanoma [14]. Of the ten genes, the Parkinson's disease-associated gene *PARK2* has been recently described as a tumor suppressor gene in glioblastoma and other malignancies [30], while *DLL1*, *HSD17B3* and *ULBP* have been reported to be associated with cancer, although not as tumor suppressors [31], [32], [33], [34], [35], [36], [37]. Experimental investigation will be required to determine if any of these ten genes performs an anti-oncogenic function in melanoma cells. The only deleted gene common to our study and those of Stark and Hayward and Gast et al. was *PTEN*, a tumor suppressor gene already known to be deleted in melanoma [7], [38].

In an alternative approach to detect recurrent events in these samples, we used a protein network-guided analysis [20], [39], [40], [41], [42], [43] to identify pathways affected by SCNA-genes in the seven melanoma cell lines. In contrast to the low level of recurrence in these melanoma samples at the individual gene level, we found that six pathways were shared by five of the samples, and four pathways (G protein, WNT, cadherin signaling and melanogenesis) were common to six (see Table 3). Several of these pathways are highly relevant to melanoma (e.g. MAPK, cadherin and FGF signaling) and have also emerged from cDNA expression studies [44], lending support to our results. G proteins transduce signals from G protein-coupled receptors (GPCRs), the largest family of membrane receptors involved in signal transduction, and whose over-expression in tumors can contribute to tumor progression, angiogenesis and metastasis [45]. Alteration of G proteins could impact the activities of GPCRs key to melanocytic cells, such as *MC1R* (melanocortin receptor), chemokine (e.g. *CXCR2*), and endothelin receptors. [46]. The recent identification of activating mutations in two G protein alpha subunits, *GNAQ* and *GNA11*, in a large proportion of uveal melanomas [47], [48], further underscores the relevance of this class of proteins to melanoma.

Although annotated as distinct pathways, WNT, cadherin signaling and melanogenesis shared six SCNA-genes in common (*FZD8* and several members of the WNT family). This may reflect interactions between these pathways, an interplay between the WNT and cadherin pathways is known to exist [49], or may be a consequence of poor pathway annotation. The cadherin pathway controls cell-adhesion and plays a role in invasion and metastasis [50]. WNT (and Hedgehog) control development and growth in the embryo; aberrant activation of their transcriptional components ultimately affects cell fate, proliferation, and migration [51], [52], [53]. The only common non-signaling pathway was melanogenesis. Melanoma develops from melanocytes, cells highly specialized in the synthesis of melanin pigment, a process that requires a complex enzymatic machinery and unique organelle structures [54]. Our pathway analysis predicted that melanoma SCNA affect melanogenesis. Loss of pigmentation in metastases compared to primary tumors is commonly observed in cutaneous melanoma, and although not completely understood, it can be brought about by different mechanisms, such as premature degradation of melanogenic proteins [55] or downregulation of *MITF* transcription program [56]. Our study suggests that SCNA may also contribute to these alterations. An unexpected pathway that emerged from our analysis, and perhaps merits further exploration, is neurotransmission. These results suggest an involvement of neuronal pathways in melanoma, possibly related to the neural crest origin of melanocytes. Lending support to this hypothesis, the metabotropic glutamate receptor *GRM1* has recently been implicated in the development of spontaneous melanoma in a mouse model, and an autocrine glutamate/GRM1 loop has been described in human melanoma [57].

Comparison of the pathways generated from SCNA-genes in our data and genes affected by copy number changes in two published datasets (Stark and Hayward [14] and Gast et al. [13]) revealed a high level of overlap, much higher than we expected based on the number of commonly affected genes (see Fig. 4). An explanation for this outcome is that different genes within the same pathway are affected in different datasets, and the commonalities are apparent only at the pathway level. The number of affected genes in a given pathway would be expected to increase with increasing sample size, and this is largely the case between our data and those of Stark and Hayward, but not in the Gast et al dataset (see Table S4). The reason for the low number of SCNA affected genes and corresponding pathways in the latter case may be the high stringency criteria employed in their analysis [13].

The angiogenesis pathway was one of ten common to all three datasets. Its up-regulation is a well-known hallmark of cancer [58], and it has long been proposed as a target for therapeutic treatment [59], [60]. Activation signals for angiogenesis include vascular endothelial growth factor (VEGF) and acidic fibroblast growth factor (FGF), and both were in our list of significantly affected pathways (see Table 3) and within our analysis of the Stark and Hayward (VEGF and FGF) and Gast et al (VEGF) datasets (see Table S4). Two genes in this pathway, *EPHA3* and *FRS2*, were designated SCNA-genes in both our dataset and in Stark and Hayward, and were annotated as amplified, in skin-derived tumors, in the Cancer Genome Project dataset [5], [61].

In our analysis *EPHA3*, an ephrin tyrosine kinase receptor, was both focally amplified and over-expressed only in LAU-Me275. However, *EPHA3* was highly over-expressed in LAU-T149D and LAU-Me246 (see Table S3) and amplified in LAU-T618A (CN = 6.4), LAU-Me235 (CN = 4) and LAU-T50B (CN = 4.2). *EPHA3* is recurrently mutated in adenocarcinoma [62], [63] and has been implicated in renal carcinoma, glioblastoma, colorectal, breast and lung cancer [63], [64], [65], [66], [67]. Mutations in *EPHA3* have been detected in melanoma [68], and several ephrin-derived peptide antigens (from *EPHA2*, *EPHA3* and *EPHB6*) can be recognized by cancer-specific cytotoxic T-cells [69], . In addition, the feasibility of specific *EPHA3* targeting has been reported [73]. These observations indicate that *EPHA3* might be a promising target for therapeutic treatment in melanoma and other cancers.

*FRS2*, fibroblast growth factor receptor substrate 2, is an adaptor that acts downstream of a limited number of receptor tyrosine kinases, in particular FGF and neurotrophin receptors, RET and ALK, and plays a major role in tumorigenesis [74]. Dey and coworkers [75] recently targeted the FGF receptors (FGFR) using tyrosine kinase inhibitors to decrease the activity of AKT and ERK kinases, inducing apoptosis in breast cancer cell lines. FGFR inhibition is highly relevant to melanoma, where autocrine stimulation via FGF2/FGFR1 constitutes a pivotal role in proliferation and survival [76]. *FRS2* has been suggested as a therapeutic target in cancer [77] and because of its downstream activities to FGFR and other receptors, it might offer new insights in melanoma treatment. In our data *FRS2* was both focally amplified and over expressed in two melanoma samples (LAU-T149D and LAU-Me275) and amplified (CN = 4) in two additional melanomas (LAU-T618A and LAU-Me235). Inspection of its amplification status in larger melanoma collections would be useful to confirm its potential role as a target of interest in melanoma.

Using SILAC, we demonstrated a global correlation between mRNA and protein levels across all samples. However, we are aware that the levels of individual proteins may not always reflect mRNA levels, and that the activity of certain proteins, and therefore the activation state of cellular pathways, can be further modulated by post-translational modifications. Further investigations, beyond the scope of the current study, will be required to address these possibilities. Such studies should include a functional assessment of the implicated pathways using various manipulations to activate or inhibit them (e.g. with siRNA or other inhibitors) to determine the role of the pathways in these melanoma cell lines.

In conclusion, we have identified SCNA-genes and pathways potentially altered in our metastatic melanoma samples and two published datasets (Stark and Hayward 2007; Gast et al., 2010) which should be investigated by screening larger tumor collections and in functional studies. Two SCNA-genes, *EPHA3* and *FRS2*, emerged from our analysis as potential therapeutic targets. These genes were replicated in our analysis of the two published melanoma collections, have been extensively studied in other cancer types, and thus might offer new insights in the treatment of malignant melanoma.

## Materials and Methods

### Melanoma samples, DNA and RNA extraction

Melanoma cell lines were established from metastases from patients with cutaneous melanoma and were used at low passage (<10). Donor matched cells were either peripheral blood lymphocytes (PBL) or Epstein-Barr virus transformed lymphoblastoid (EBV) cell lines. EBV cell lines were karyotyped to ensure genome stability and diploidy. Approval to use these samples for this project was given by the CHUV (Centre Hospitalier Universitaire Vaudois) ethical committee for clinical research. Melanoma cell lines were cultured conditions in RPMI-1640 medium supplemented with 10% fetal calf serum (FCS), and no antibiotics. Human foreskin melanocytes were grown in HAM-F10 medium supplemented with 2% FCS, 5% MelanoMax supplement (Gentaur, Belgium) and 6 mM HEPES. EBV cell lines were cultured in IMDM/10% FCS medium. All cultures were without mycoplasma. DNA (Gentra kit, Qiagen) and RNA (guanidinium/cesium chloride gradient) isolation and karyotype preparations were performed from parallel cultures.

### Cytogenetic and FISH analysis

Cytogenetic (GTG-banding) and fluorescence in situ hybridization (FISH) metaphase analyses of melanoma cell lines were performed using standard protocols. We performed 15 spreads for each melanoma cell line, except for Lau-Me246 [14] and Lau-Me275 [21]. Dual color FISH was done using a commercially available set consisting of a locus-specific *MDM2* combined with a chromosome 12 centromeric probe (Kreatech Poseidon FISH probe) to distinguish aneuploidy of chromosome 12 and specific locus loss or gain. Chromosomes with homogeneously staining region (HSR) were identified with the analysis of FISH metaphases in inverted digital images. Copy number estimation of HSR was done using FISH interphases.

### Comparative genomic arrays (CGH)

CGH arrays were processed according to the manufacturer's protocol (Agilent Technologies, Inc.) and as described in Martinet et al. [78].

The normalization and detection of copy number aberration is detailed in Methods S1. In brief, signal intensities were normalized using three independent normalization schemes: Loess [79]; PopLowess [80]; and the statistical framework from Chen et al. [15].

Then probe-level data were segmented using Circular Binary Segmentation [81], [82] and attributed a discrete copy number to segments using three independent methods: 1) a naive scoring-based approach, where outliers relative to the chromosomal baseline are detected using a non-parametric score; 2) the MergeLevels method [83] and 3) our own classification algorithm based on Gaussian Mixture Model which models the observed distribution of intensity ratios as a combination of Gaussian distributions that can be subsequently classified into deletion (CN<2), copy neutral event (CN = 2), duplication and amplification (CN = 3 and CN≥4) (see Fig. S3).

### Single Nucleotide polymorphism arrays (SNP)

#### Illumina 1M SNP arrays

Genomic DNA from each of the 7 melanoma and their matched normal cells (either EBV cell line or PBL); as well as two control melanocytes were genotyped on the Illumina Infinium Human1M-Duo arrays. Aliquots of DNA (30 µl at 50 ng/µl) for each sample were processed according to the manufacturer's protocol (Infinium HD Gemini protocol). Subsequently we used the OverUnder algorithm [16] to correct the hybridization log ratios for polyploidy and to attribute a continuous copy number value to each SNP. We estimated that the window size parameter set to 201 SNPs, gave the highest reproducibility between technical replicates (see Fig. S5A and S5B).

#### Affymetrix 6.0 arrays

As part of the technical replicate design, we analyzed LAU-Me275 on Affymetrix 6.0 SNP arrays. The experiment was performed in accordance with the manufacturer's instructions. Normalization and copy number prediction were done using the PICNIC algorithm [84].

### Transcriptome sequencing

The transcriptome from all seven melanoma as well as a pool of two melanocytes was sequenced using the Roche 454 Titanium technology. mRNA isolation and cDNA preparation were performed following the protocol used by Bainbridge et al. [85], with some modifications (See Methods S1). 3–5 µg of cDNA were used for 454 libraries preparation, according to manufacturer's protocol. All experiments produced about 1M single end reads, with a median length of 367 nucleotides (interquartile range 265–436). We derived transcript tag counts using our own published methodology [86] (see also Methods S1). Using tag counts from the pool of melanocytes, we were able to derive a ratio of expression for each melanoma with respect to these control melanocytes.

### Detection of somatic copy number alterations with altered expression (SCNA-genes)

We computed the median copy number at each Refseq gene. To overcome density limitations, we included SNPs that were within 2 kb of the gene boundaries. For CGH arrays, we included probes within 3 kb of the gene boundaries. We defined SCNA-genes as follows. A gene was flagged as within a focal amplification when its CN, as computed from SNP arrays, was ≥4, the difference in CN relative to the chromosomal arm was ≥1, the gene was diploid (CN = 2) in the matched control cell line, and the expression in the melanoma cell line was at least 2-fold greater than that in the control melanocytes. For deletions, a gene needed to have CN<2, as detected by CGH, without expression in the melanoma cell line and CN = 2 with detected expression in the melanocytes.

### Protein network-guided analysis of SCNA

A non-redundant human protein interaction network was generated by combining iRefseq [87] and Pathway Commons [88] protein interaction databases with functional interactions from Panther pathways [89]. The resulting network has 21,876 nodes and 376,528 edges and combines interaction data from 15 primary protein interaction databases (BIND, BioGRID, CORUM, DIP, HPRD, IntAct, MINT, MPact, MPPI, OPHID, Reactome, HumanCyc, Cancer Cell Map, IMID and NCI/Nature pathway interaction database). Mapping between gene names and their protein Uniprot IDs was downloaded from the HGNC website (www.genenames.org). Among the 1710 (unique) SCNA-genes, 104 did not have a UniProt ID and 43 could not be mapped onto the network. Using a walk trap community algorithm and permutation approaches (with n = 1000), we were able to extract clusters of proteins from the network (Details are available in Methods S1).

### Pathway analysis

Significance of overlap between the modules and pathways from Panther, Kegg and MSigDB [89], [90], [91] was calculated with an hypergeometric test, P-values were corrected for multiple comparisons by calculating the false discovery rate using the Benjamini and Hochberg procedure [92].

To reduce the redundancy present in pathway database annotation, we first combined pathways that contained the same SCNA-genes from the same melanoma cell lines, then we reviewed the pathway list to either remove redundancies or un-merge unrelated pathways affected by similar genes. We also excluded KEGG “cancer” annotated pathways.

### Proteomic analysis

#### SILAC

Cell growth conditions and labeling are detailed in Methods S1. LC-MS/MS data were analyzed using Mascot 2.2 (Matrix Science, London, UK) for database search and MaxQuant 1.0.13.13 for peak list export from raw data and quantitative analysis [93]. Database search was carried out against the IPI human database (version 3.52, [94]) including decoy sequences and a list of common contaminants. A maximum false discovery rate of 1% was used for protein identification.

A total of 5522 proteins were detected by the SILAC experiment. We excluded 55 proteins that matched the list of common contaminants. To check whether culture condition (with the heavy isotope) could influence the protein quantification, we performed a self-self experiment with LAU-Me275. Although no significant bias was found (see Fig. S9), we excluded 79 proteins that were outliers in this experiment. Next, we checked the number of peptides detected per protein (see Fig. S10). Since protein quantification is not accurate when only few peptides are observed, we excluded 2,134 proteins that had less than three peptides and 42 with missing values in all seven experiments. This led to a cleaned dataset of 3,212 proteins; 2,922 of them could be remapped to Refseq genes using their Uniprot ID and were used for subsequent comparison with RNA-seq data.

#### Comparison with RNA-seq data

For comparison, we normalized the RNA seq tag count in the same manner as the SILAC data (i.e. we expressed the transcript tag count from LAU-Me275 with respect to all other samples, including the control melanocytes). Next, we computed the Spearman correlation coefficient between the expression and protein log_2_ ratios (see Fig. S11). P values to test for significantly positive correlation were estimated using large-sample approximations (as implemented by the ‘corr’ function in MATLAB).

### Accession number

Microarray and sequencing data were deposited in NCBI GEO and are available under accession number GSE23056.

## Supporting Information

Figure S1 **CGH hybridization ratio in a tetraploid region in LAU-Me275.** Each plot shows the hybridization log_2_ ratio at each CGH probe (in gray) obtained using three normalization methods. Ridge refers to the framework from Chen et al. Red segments were obtained using Circular Binary Segmentation. Karyotype analysis of LAU-Me275 revealed 11q amplification (CN≥4), so the expected CGH log ratio would be two. Here the ratios obtained from three different normalizations failed to reflect the amplification (both Loess and Ridge were close to 0; PopLowess was close to 0.6 indicating 3 copies).
(DOC)

Figure S2 **Correlation between replicates using different normalization schemes.** The scatter plots illustrate the correlation at each CGH probe between two replicates. The heatmaps show the correlation for each pairs of replicates. The normalization method is indicated in each plot title. RIDGE refers to the framework from Chen et al.
(DOC)

Figure S3 **Gaussian Mixtures identified in four replicates from LAU-Me275.** Each histogram shows the distribution of CBS segment log2 ratios, colors highlight the Gaussian components. The number of components identified and the Bayes Information Criterion are indicated in each figure title.
(DOC)

Figure S4 **Comparison of CNV detection algorithm on CGH data.** Panels are, from left to right: a melanoma with large deletions (LAU-Me280); a melanoma with large amplifications (LAU-Me275); and a control EBV cell line (male) hybridized using a pool of female references. From top to bottom: CNV classification (following CBS segmentation) using 1) Gaussian Mixture Model (GMM), 2) MergeLevels, 3) the scoring-based approach. Each dot corresponds to a CGH probe with its genomic position on the X axis and its log2 ratio of hybridization on the Y axis. Colors indicate the copy number state: orange< = 1 copy gray = 2 copies, cyan = 3 copies and dark blue more than 3 copies. For the scoring approach distinction is made between 1 copy (orange) and 0 copy (red).
(DOC)

Figure S5 **Optimization of Illumina analysis and comparison with Affymetrix prediction in LAU-Me275.** **A.** Pearson correlation between SNP CN, as a function of OverUnder window size. **B.** Copy number concordance at each SNP for different window sizes of OverUnder. Colors indicate window size parameters, the bar height indicates the total number of SNPs (in log10 scale) found in one replicate. The gray bar indicates the intersection between two technical replicates. The percentage of concordance (number of SNPs found with the same copy number bin in both replicates/total number of SNPs from this given copy number bin in the first replicate) is shown on top of each bar. **C.** Copy number prediction on chromosome 1 using OverUnder with a window size of 201 SNPs. **D.** Copy number prediction on chromosome 1 using an Affymetrix 6.0 array (with the PICNIC algorithm).
(DOC)

Figure S6 **Copy number analysis using Illumina SNP arrays.** DNA from LAU-Me275 was hybridized to Illumina SNP arrays, and the data were analyzed using the method of Attiyeh et al. The top panel shows genome-wide copy number: dark blue indicates more than three copies; cyan:three copies; gray:copy neutral; orange: deletion. Subsequent panels show chromosome 7 with, from top to bottom: Hybridization log2 ratio; B allele frequency; and copy number prediction.
(DOC)

Figure S7 **Intersection between CGH and SNP predictions.** **A.** Intersection between CGH and SNP predictions for genes with more than 4 copies. **B.** Intersection for genes within deletions. **C.** Intersection for genes within deletions for which expression was not detected.
(DOC)

Figure S8 **Copy number prediction from CGH and SNP arrays, LOH prediction from SNP arrays.**
(PDF)

Figure S9 **Boxplots of SILAC heavy/light normalized log2 ratios.** In all experiments, LAU-Me275 was labeled with the heavy isotope; the unlabeled sample is indicated in the boxplot label on the X axis. ‘NHM’ refers to the pool of normal melanocytes and ‘self-self’ to a control experiment using only LAU-Me275 to check for any bias due to the label/no label culture conditions (no significant bias was detected).
(DOC)

Figure S10 **Histogram of unique peptides identified per protein in the SILAC data.**
(DOC)

Figure S11 **Correlation between mRNA expression and protein levels.** Both SILAC and RNA seq log2 ratios are expressed for LAU-Me275 with respect to the sample indicated in each plot title. Spearman Rho correlation coefficient is also indicated in the title. In all experiments, the correlation is significantly positive (p<0.001).
(DOC)

Table S1 **Count of genes affected by SCNA.**
(DOC)

Table S2 **Processed list of SCNA genes in all seven melanoma cell lines.**
(XLS)

Table S3 **Genomic and transcriptomic data for SCNA-genes in all seven melanoma cell lines.**
(XLS)

Table S4 **List of pathways significantly enriched in SCNA, and pathway comparison between three melanoma datasets.**
(XLS)

Table S5 **List of genes contributing to pathway enrichment in three melanoma datasets.**
(XLS)

Methods S1 (DOC)
