# Supplementary material for: Network-Guided Analysis of Genes with Altered Somatic Copy Number and Gene Expression Reveals Pathways Commonly Perturbed in Metastatic Melanoma
Source: PLoS One. 2011 Apr 8;6(4):e18369. doi: 10.1371/journal.pone.0018369 (PMC3072964; doi:10.1371/journal.pone.0018369)
Supplement: Figure S1 — CGH hybridization ratio in a tetraploid region in LAU-Me275. Each plot shows the hybridization log2 ratio at each CGH probe (in gray) obtained using three normalization methods. Ridge refers to the framework from Chen et al. Red segments were obtained using Circular Binary Segmentation. Karyotype analysis of LAU-Me275 revealed 11q amplification (CN≥4), so the expected CGH log ratio would be two. Here the ratios obtained from three different normalizations failed to reflect the amplification (both Loess and Ridge were close to 0; PopLowess was close to 0.6 indicating 3 copies). (DOC) [file pone.0018369.s001.doc]

**
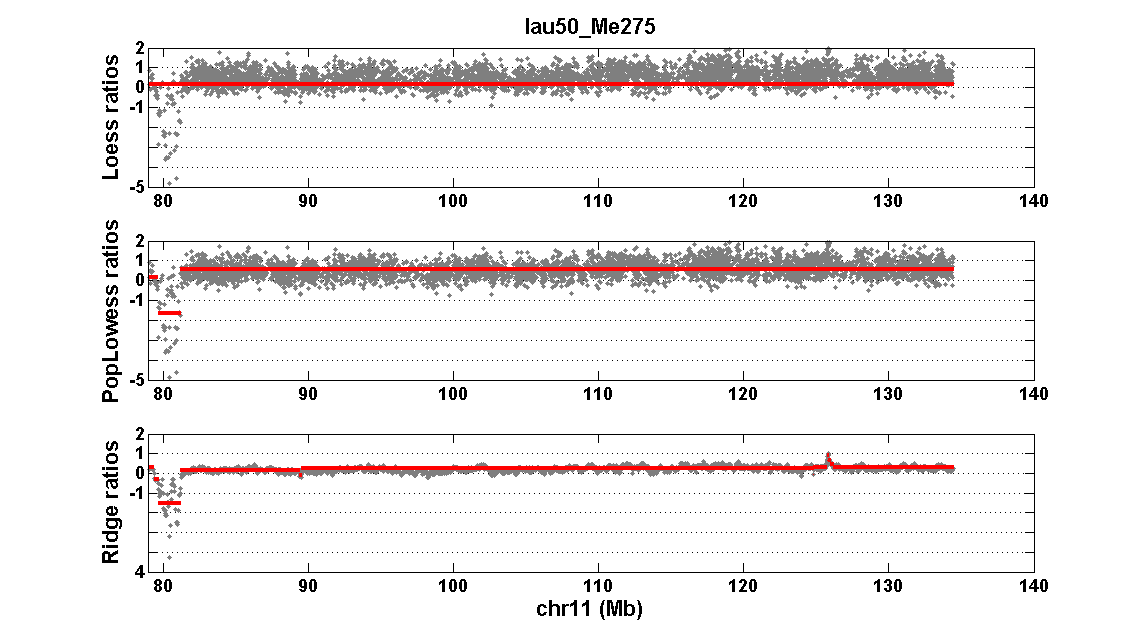
**

Figure S1. CGH hybridization ratio in a tetraploid region in LAU-Me275.

Each plot shows the hybridization log_2_ ratio at each CGH probe (in gray) obtained using three normalization methods. Ridge refers to the framework from Chen et al. Red segments were obtained using Circular Binary Segmentation. Karyotype analysis of LAU-Me275 revealed 11q amplification (CN≥4), so the expected CGH log ratio would be two. Here the ratios obtained from three different normalizations failed to reflect the amplification (both Loess and Ridge were close to 0; PopLowess was close to 0.6 indicating 3 copies).
