# Supplementary material for: Network-Guided Analysis of Genes with Altered Somatic Copy Number and Gene Expression Reveals Pathways Commonly Perturbed in Metastatic Melanoma
Source: PLoS One. 2011 Apr 8;6(4):e18369. doi: 10.1371/journal.pone.0018369 (PMC3072964; doi:10.1371/journal.pone.0018369)
Supplement: Figure S2 — Correlation between replicates using different normalization schemes. The scatter plots illustrate the correlation at each CGH probe between two replicates. The heatmaps show the correlation for each pairs of replicates. The normalization method is indicated in each plot title. RIDGE refers to the framework from Chen et al. (DOC) [file pone.0018369.s002.doc]

| LOESS  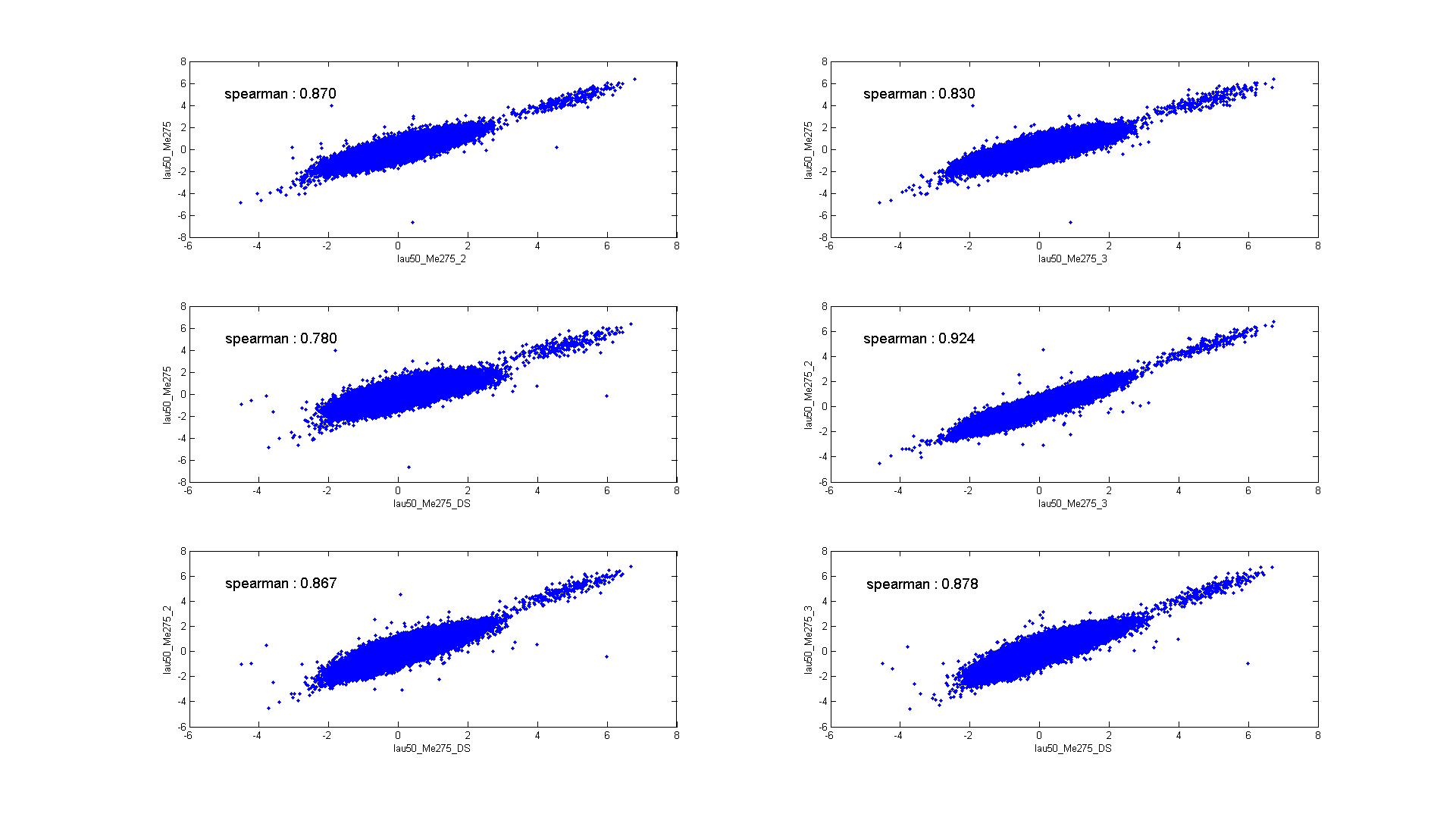 | 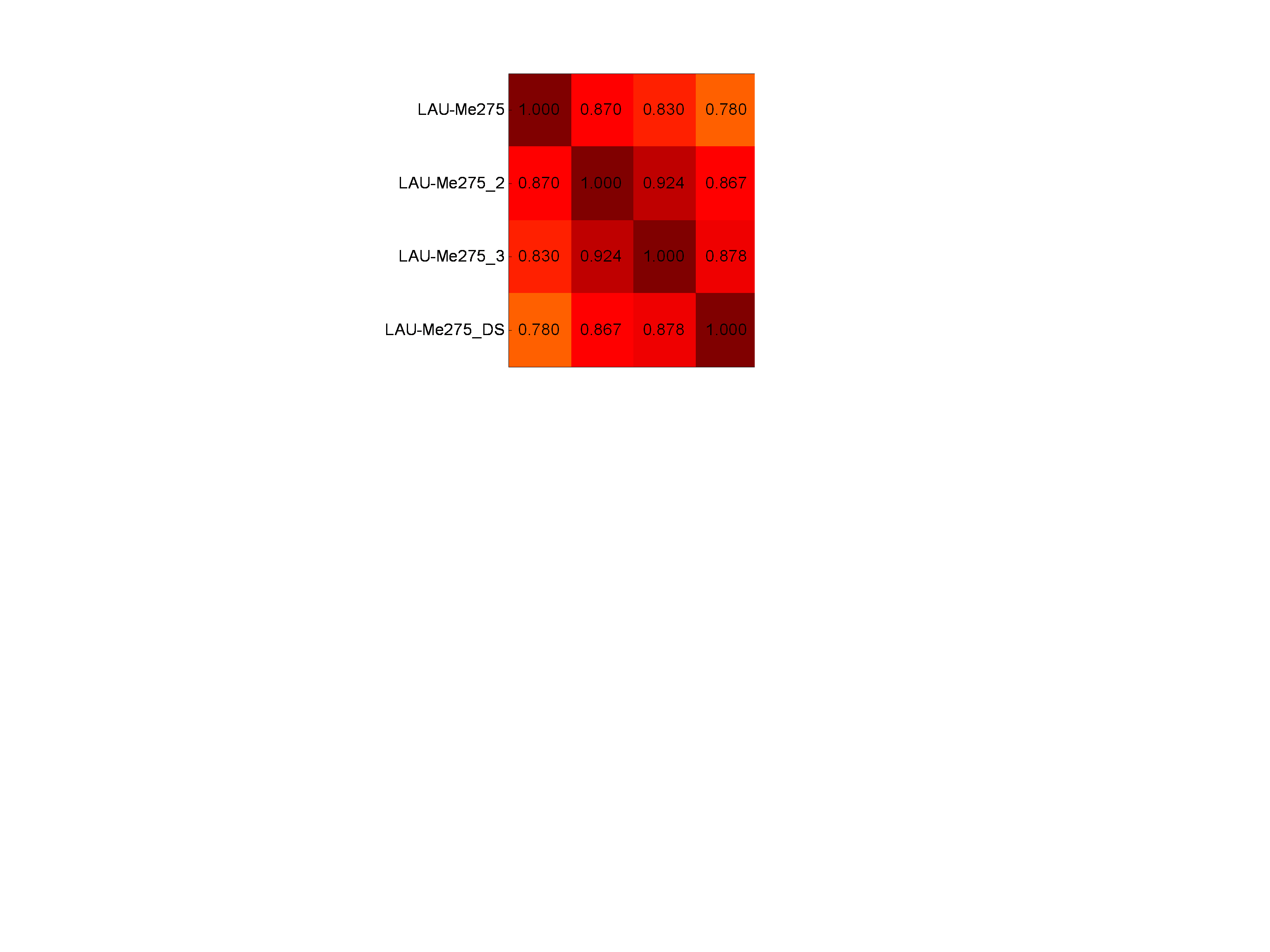 |
| --- | --- |
| POPLOWESS  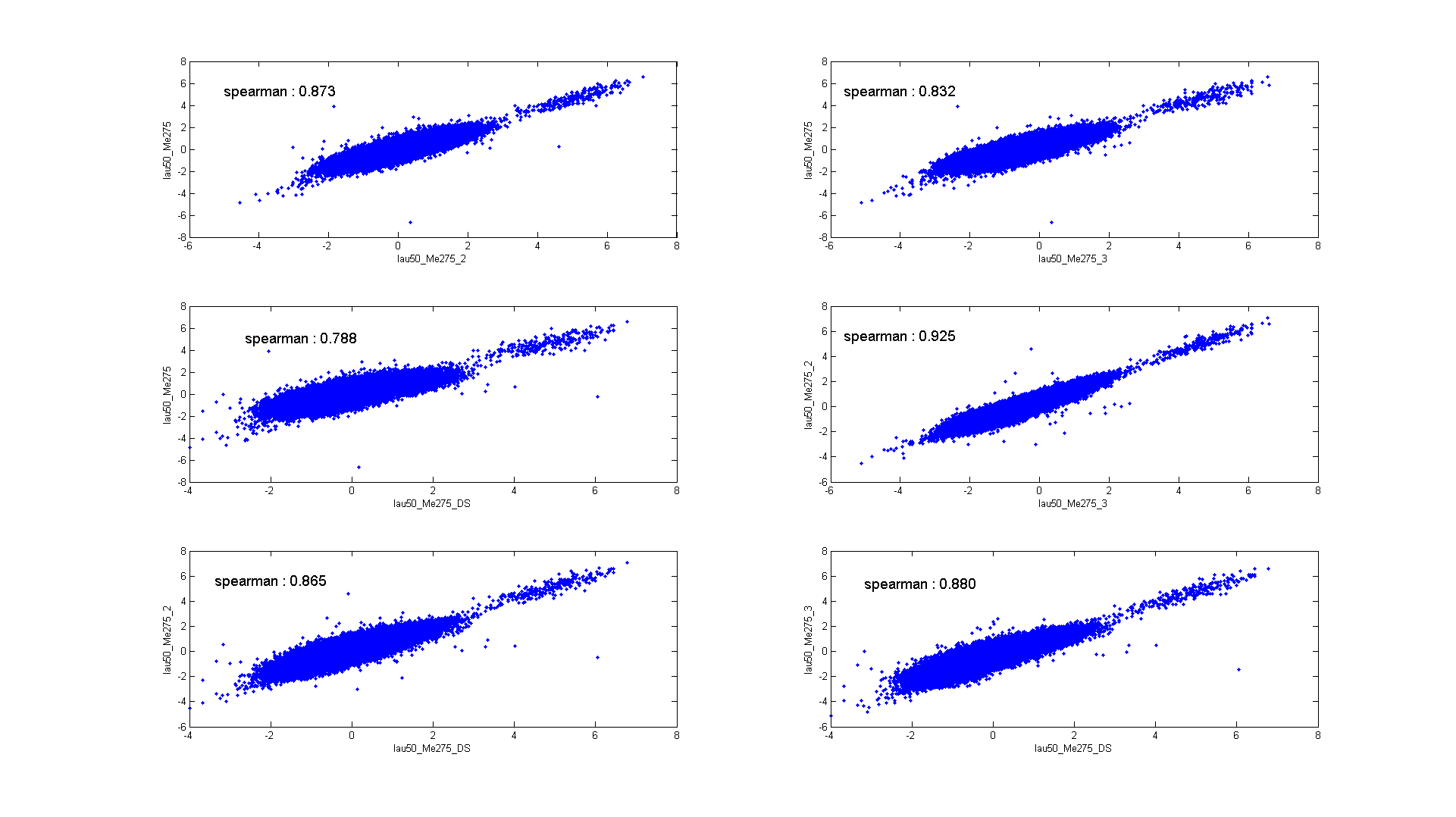 | 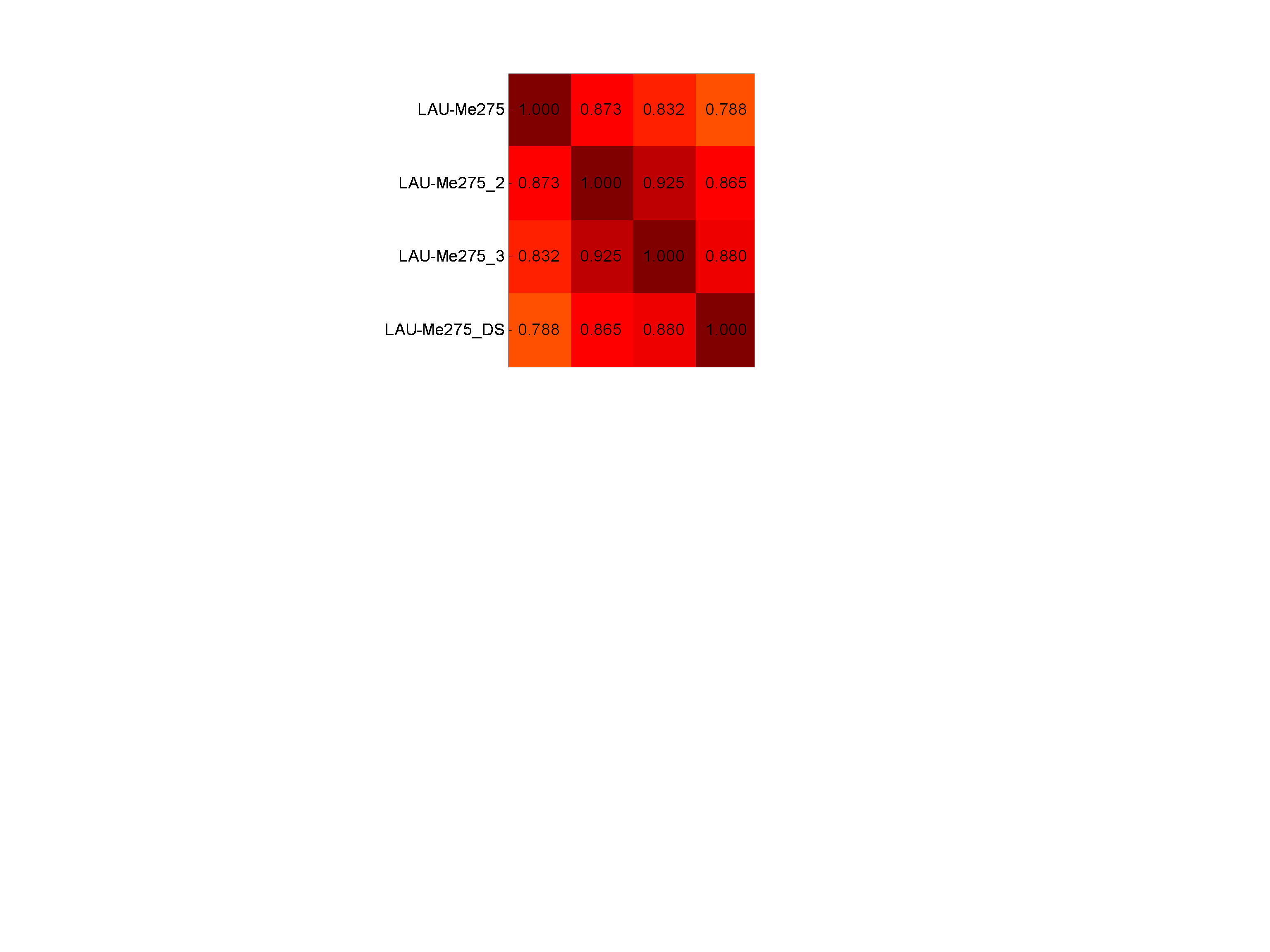 |
| RIDGE  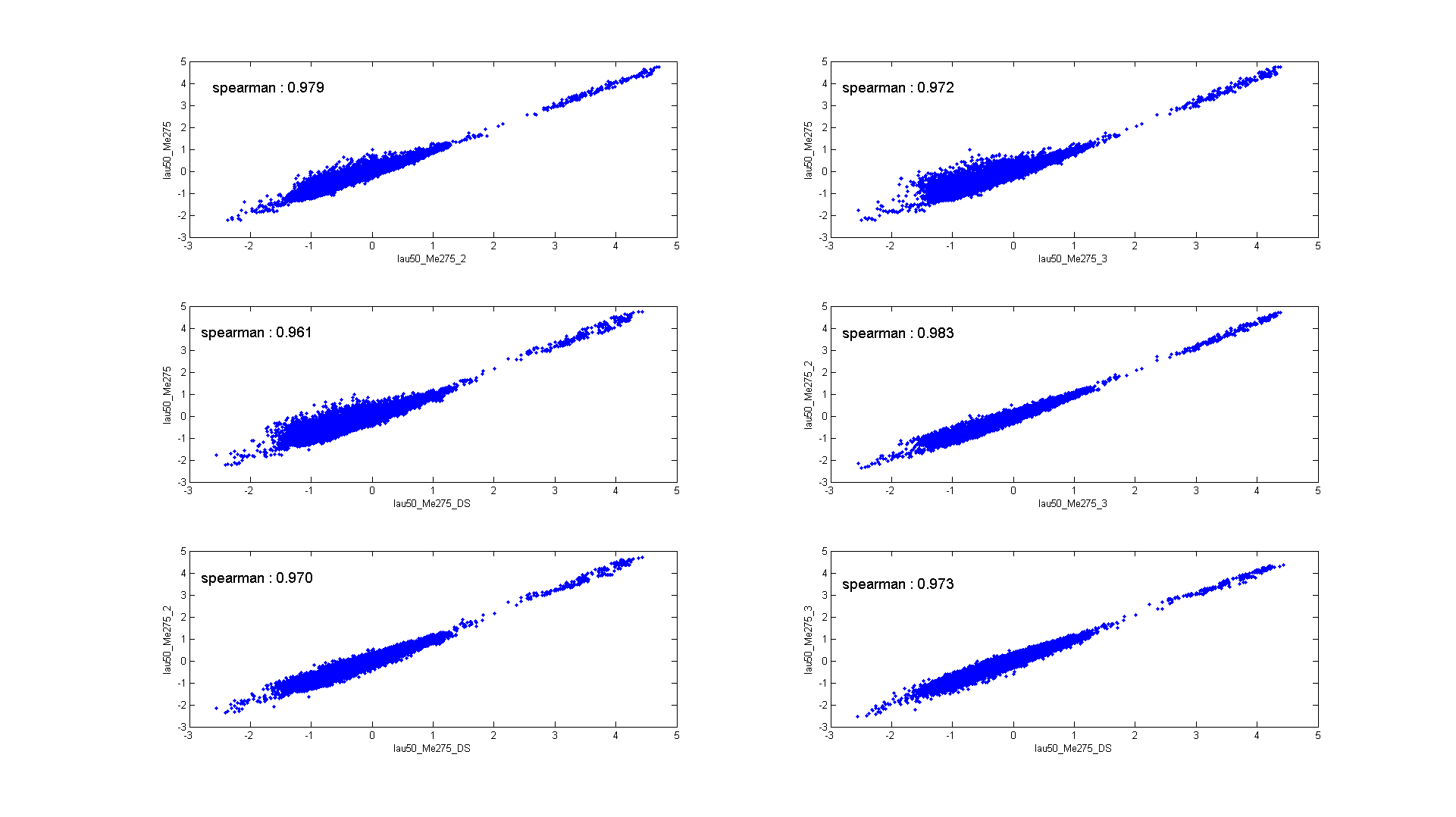 | 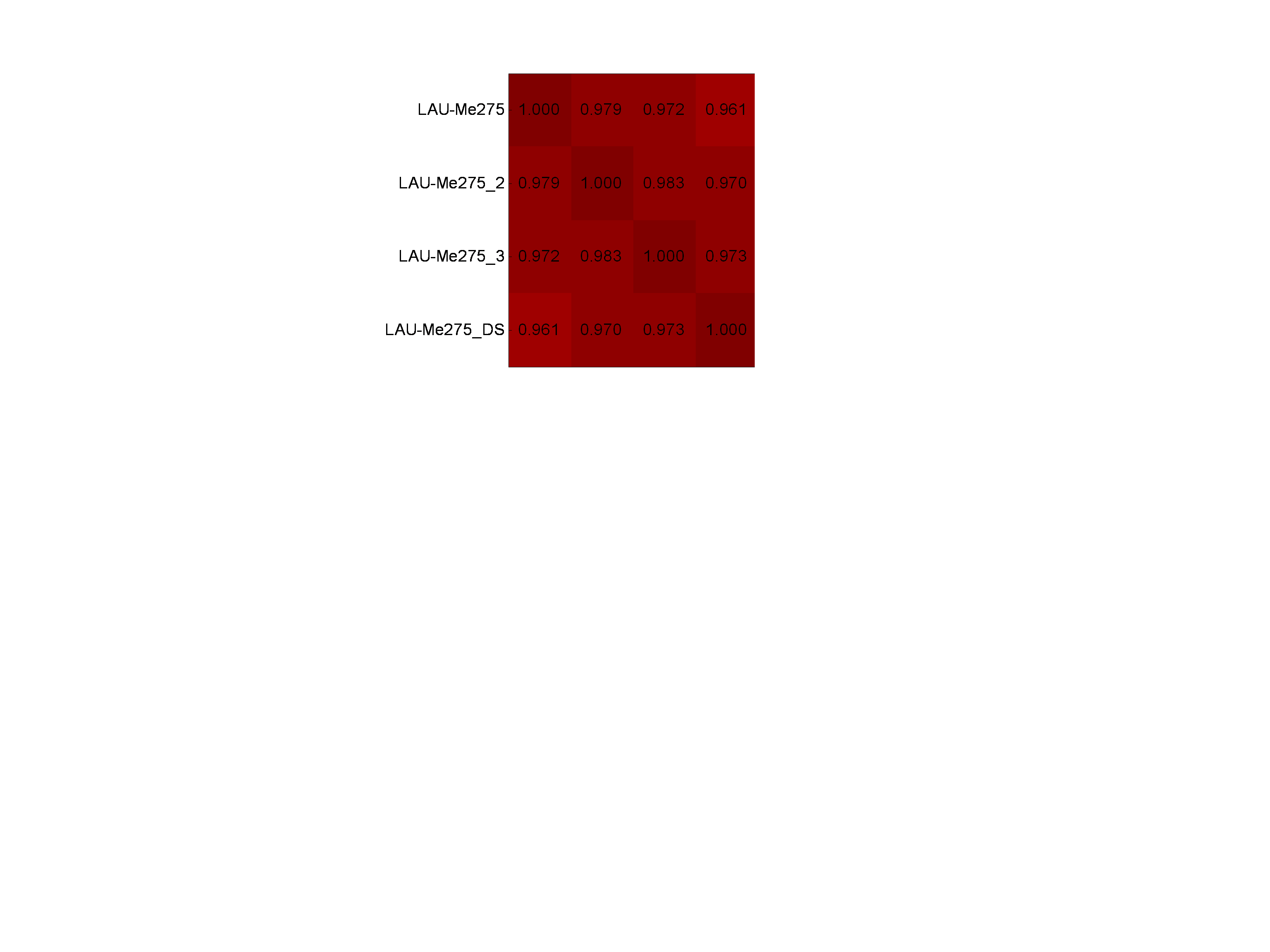 |

Figure S1. Correlation between replicates using different normalization schemes.

The scatter plots illustrate the correlation at each CGH probe between two replicates. The heatmaps show the correlation for each pairs of replicates. The normalization method is indicated in each plot title. RIDGE refers to the framework from Chen et al.
