# Supplementary material for: Network-Guided Analysis of Genes with Altered Somatic Copy Number and Gene Expression Reveals Pathways Commonly Perturbed in Metastatic Melanoma
Source: PLoS One. 2011 Apr 8;6(4):e18369. doi: 10.1371/journal.pone.0018369 (PMC3072964; doi:10.1371/journal.pone.0018369)
Supplement: Figure S3 — Gaussian Mixtures identified in four replicates from LAU-Me275. Each histogram shows the distribution of CBS segment log2 ratios, colors highlight the Gaussian components. The number of components identified and the Bayes Information Criterion are indicated in each figure title. (DOC) [file pone.0018369.s003.doc]

|  |  |
| --- | --- |
|  |  |

Figure S1. Gaussian Mixtures identified in four replicates from LAU-Me275.

Each histogram shows the distribution of CBS segment log2 ratios, colors highlight the Gaussian components. The number of components identified and the Bayes Information Criterion are indicated in each figure title.
