# Supplementary material for: Network-Guided Analysis of Genes with Altered Somatic Copy Number and Gene Expression Reveals Pathways Commonly Perturbed in Metastatic Melanoma
Source: PLoS One. 2011 Apr 8;6(4):e18369. doi: 10.1371/journal.pone.0018369 (PMC3072964; doi:10.1371/journal.pone.0018369)
Supplement: Figure S4 — Comparison of CNV detection algorithm on CGH data. Panels are, from left to right: a melanoma with large deletions (LAU-Me280); a melanoma with large amplifications (LAU-Me275); and a control EBV cell line (male) hybridized using a pool of female references. From top to bottom: CNV classification (following CBS segmentation) using 1) Gaussian Mixture Model (GMM), 2) MergeLevels, 3) the scoring-based approach. Each dot corresponds to a CGH probe with its genomic position on the X axis and its log2 ratio of hybridization on the Y axis. Colors indicate the copy number state: orange< = 1 copy gray = 2 copies, cyan = 3 copies and dark blue more than 3 copies. For the scoring approach distinction is made between 1 copy (orange) and 0 copy (red). (DOC) [file pone.0018369.s004.doc]

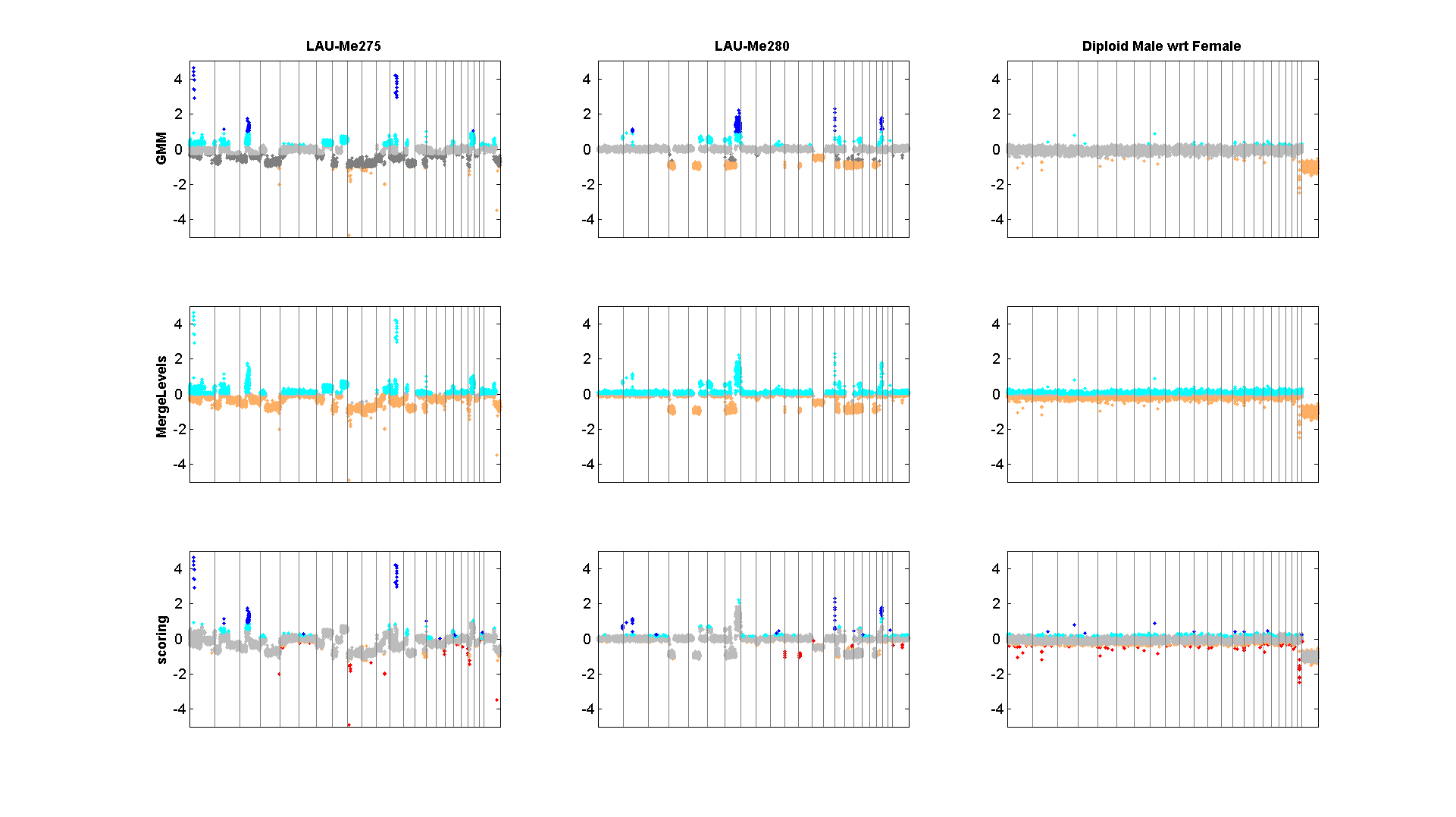


Figure S1. Comparison of CNV detection algorithm on CGH data.

Panels are, from left to right: a melanoma with large deletions (LAU-Me280); a melanoma with large amplifications (LAU-Me275); and a control EBV cell line (male) hybridized using a pool of female references. From top to bottom: CNV classification (following CBS segmentation) using 1) Gaussian Mixture Model (GMM), 2) MergeLevels, 3) the scoring-based approach. Each dot corresponds to a CGH probe with its genomic position on the X axis and its log2 ratio of hybridization on the Y axis. Colors indicate the copy number state : orange <= 1 copy gray = 2 copies, cyan = 3 copies and dark blue more than 3 copies. For the scoring approach distinction is made between 1 copy (orange) and 0 copy (red).
