# Supplementary material for: Network-Guided Analysis of Genes with Altered Somatic Copy Number and Gene Expression Reveals Pathways Commonly Perturbed in Metastatic Melanoma
Source: PLoS One. 2011 Apr 8;6(4):e18369. doi: 10.1371/journal.pone.0018369 (PMC3072964; doi:10.1371/journal.pone.0018369)
Supplement: Figure S5 — Optimization of Illumina analysis and comparison with Affymetrix prediction in LAU-Me275. A. Pearson correlation between SNP CN, as a function of OverUnder window size. B. Copy number concordance at each SNP for different window sizes of OverUnder. Colors indicate window size parameters, the bar height indicates the total number of SNPs (in log10 scale) found in one replicate. The gray bar indicates the intersection between two technical replicates. The percentage of concordance (number of SNPs found with the same copy number bin in both replicates/total number of SNPs from this given copy number bin in the first replicate) is shown on top of each bar. C. Copy number prediction on chromosome 1 using OverUnder with a window size of 201 SNPs. D. Copy number prediction on chromosome 1 using an Affymetrix 6.0 array (with the PICNIC algorithm). (DOC) [file pone.0018369.s005.doc]

| 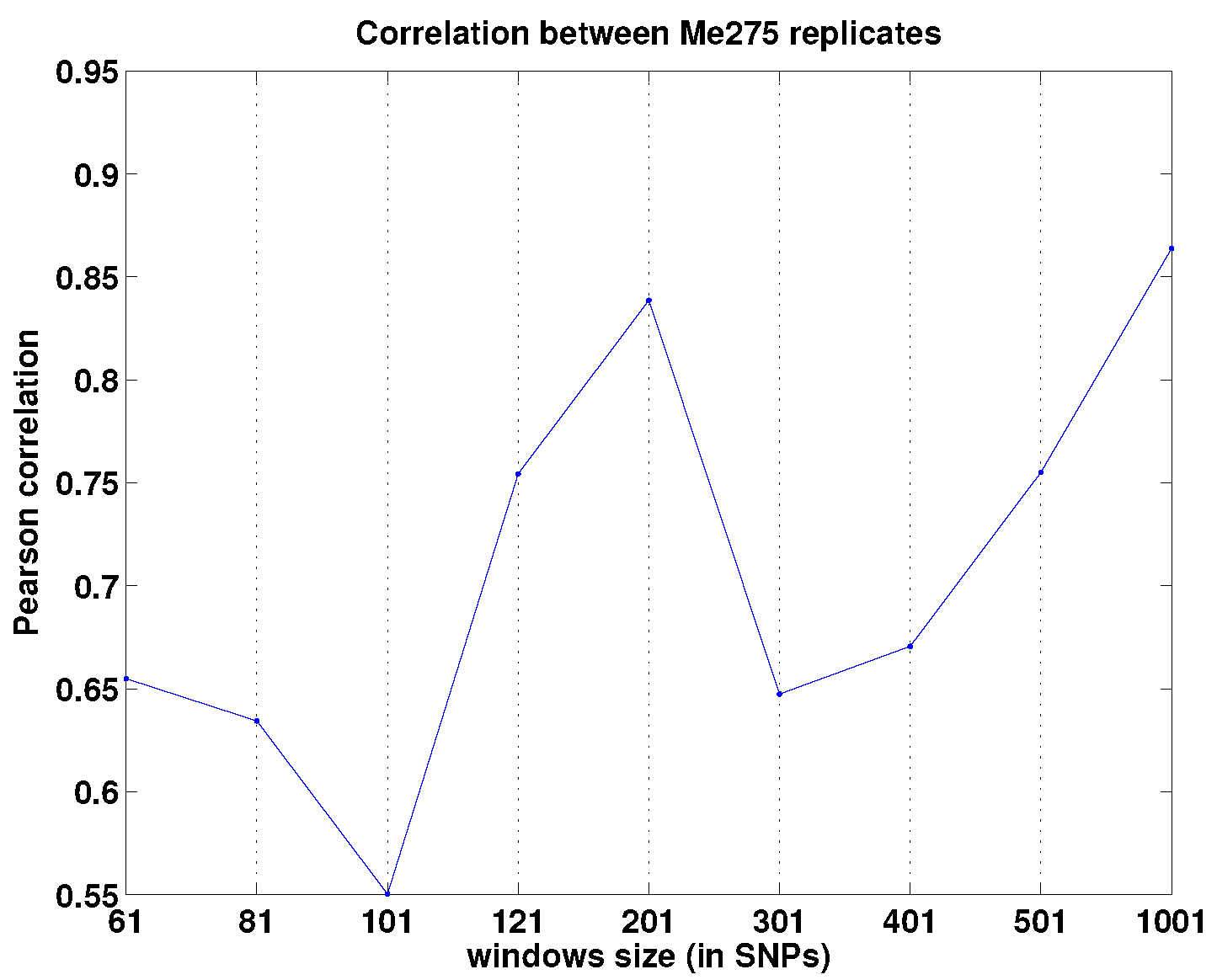  **A**  **B** | **C**  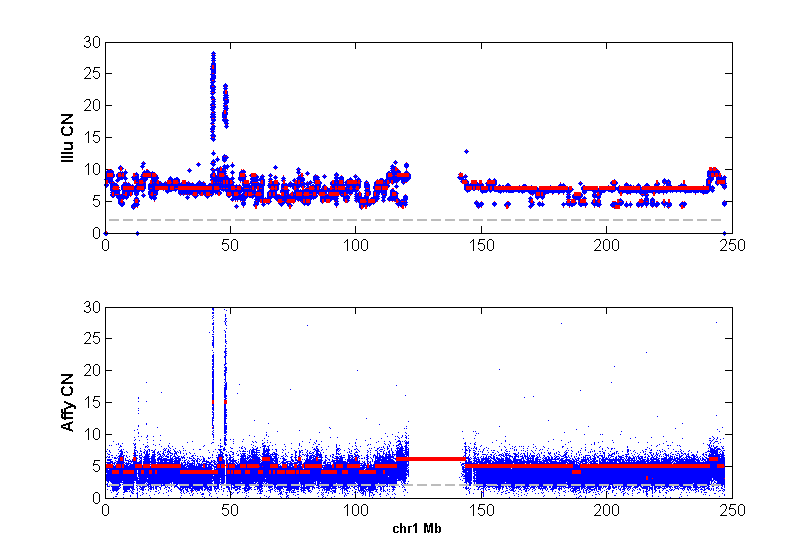  **D** |
| --- | --- |
|  | 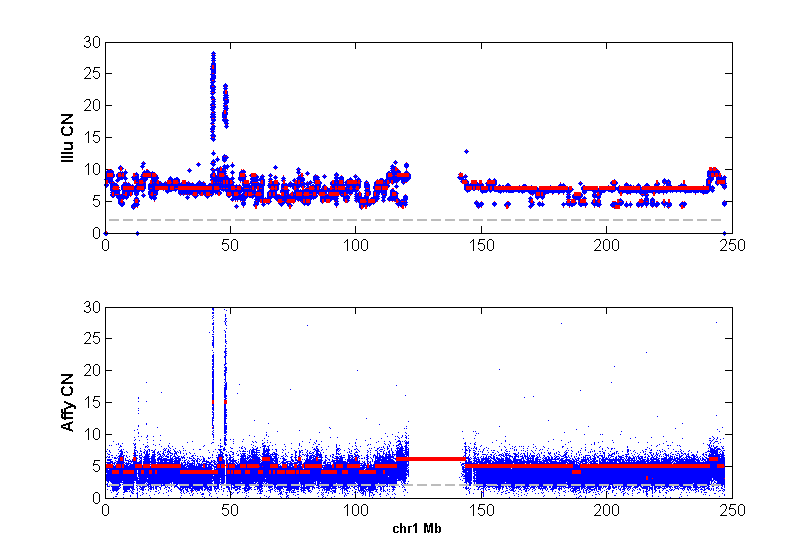 |

Figure S5. Optimization of Illumina analysis and comparison with Affymetrix prediction in LAU-Me275.

**A.** Pearson correlation between SNP CN, as a function of OverUnder window size. **B.** Copy number concordance at each SNP for different window sizes of OverUnder. Colors indicate window size parameters, the bar height indicates the total number of SNPs (in log10 scale) found in one replicate. The gray bar indicates the intersection between two technical replicates. The percentage of concordance (number of SNPs found with the same copy number bin in both replicates / total number of SNPs from this given copy number bin in the first replicate) is shown on top of each bar. **C.** Copy number prediction on chromosome 1 using OverUnder with a window size of 201 SNPs. **D.** Copy number prediction on chromosome 1 using an Affymetrix 6.0 array (with the PICNIC algorithm).
