# Supplementary material for: Network-Guided Analysis of Genes with Altered Somatic Copy Number and Gene Expression Reveals Pathways Commonly Perturbed in Metastatic Melanoma
Source: PLoS One. 2011 Apr 8;6(4):e18369. doi: 10.1371/journal.pone.0018369 (PMC3072964; doi:10.1371/journal.pone.0018369)
Supplement: Figure S6 — Copy number analysis using Illumina SNP arrays. DNA from LAU-Me275 was hybridized to Illumina SNP arrays, and the data were analyzed using the method of Attiyeh et al. The top panel shows genome-wide copy number: dark blue indicates more than three copies; cyan:three copies; gray:copy neutral; orange: deletion. Subsequent panels show chromosome 7 with, from top to bottom: Hybridization log2 ratio; B allele frequency; and copy number prediction. (DOC) [file pone.0018369.s006.doc]

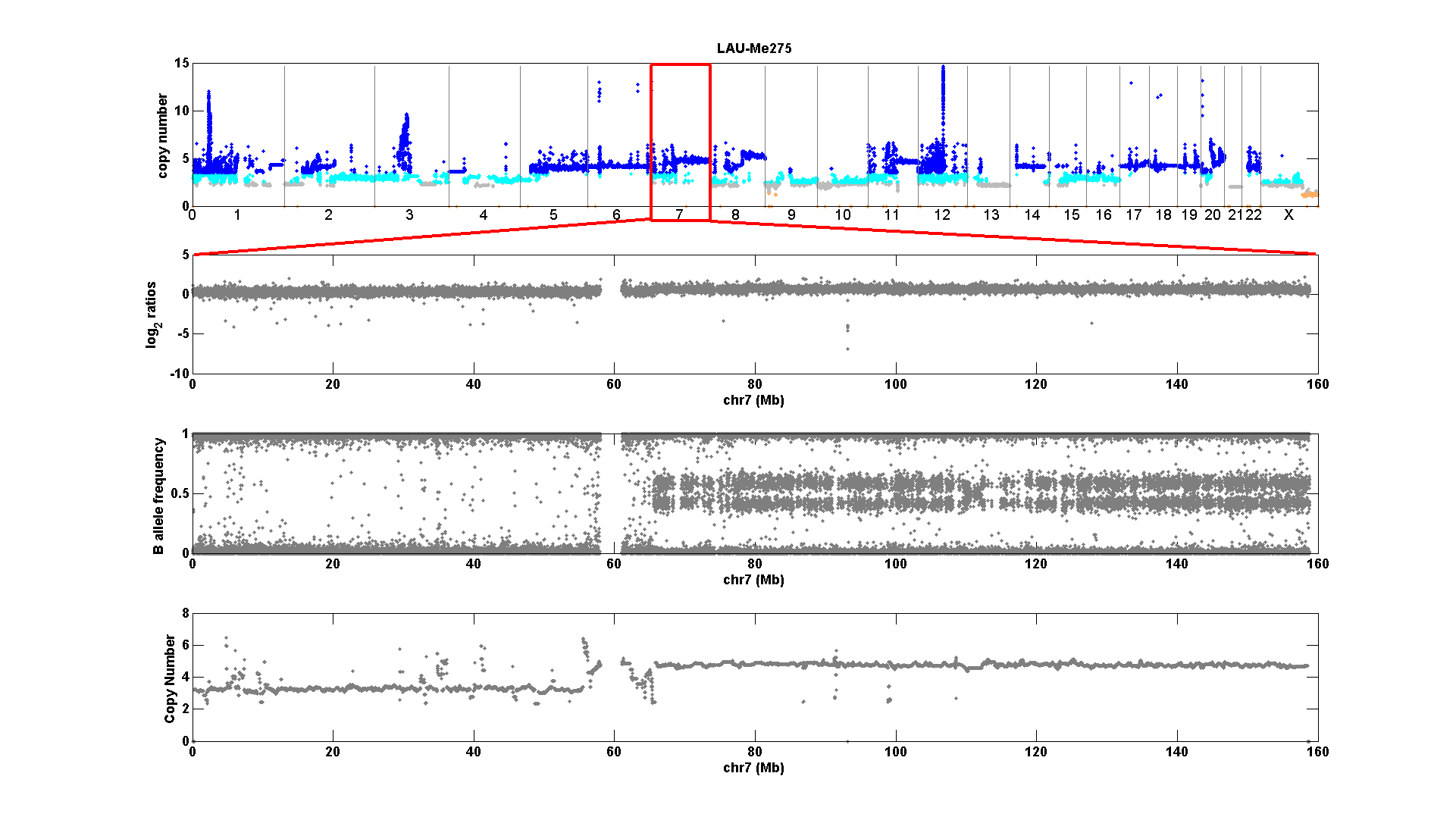


Figure S6. Copy number analysis using Illumina SNP arrays.

DNA from LAU-Me275 was hybridized to Illumina SNP arrays, and the data were analyzed using the method of Attiyeh et al. The top panel shows genome-wide copy number: dark blue indicates more than three copies; cyan:three copies; gray:copy neutral; orange : deletion. Subsequent panels show chromosome 7 with, from top to bottom: Hybridization log2 ratio; B allele frequency; and copy number prediction.
