# Supplementary material for: Network-Guided Analysis of Genes with Altered Somatic Copy Number and Gene Expression Reveals Pathways Commonly Perturbed in Metastatic Melanoma
Source: PLoS One. 2011 Apr 8;6(4):e18369. doi: 10.1371/journal.pone.0018369 (PMC3072964; doi:10.1371/journal.pone.0018369)
Supplement: Figure S7 — Intersection between CGH and SNP predictions. A. Intersection between CGH and SNP predictions for genes with more than 4 copies. B. Intersection for genes within deletions. C. Intersection for genes within deletions for which expression was not detected. (DOC) [file pone.0018369.s007.doc]

**A**

**C**

**B**

| 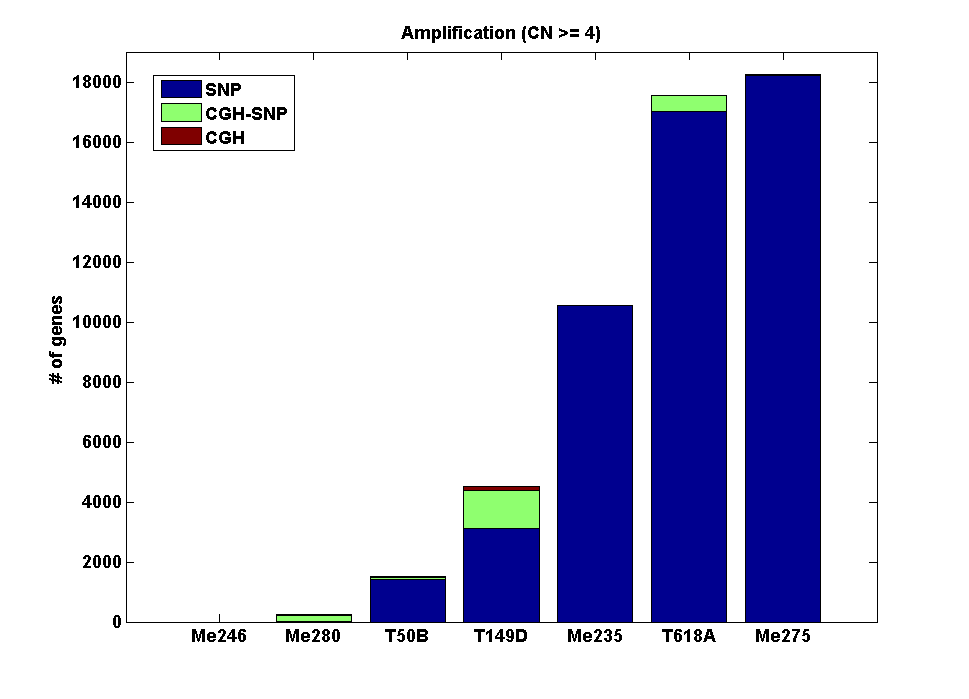 | 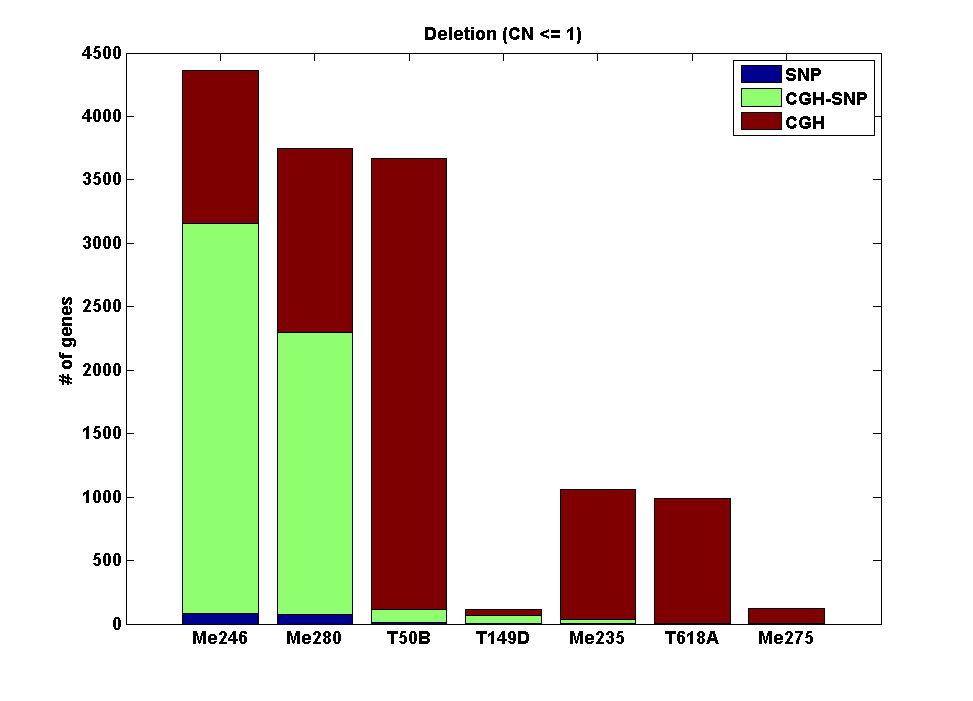 | 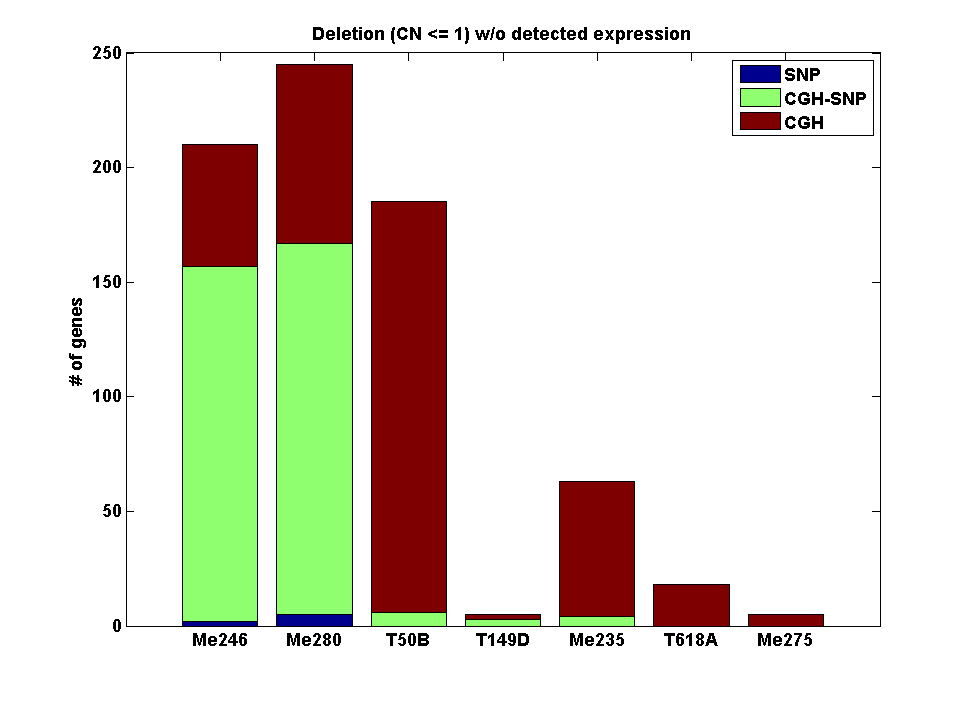 |
| --- | --- | --- |

Figure S7. Intersection between CGH and SNP predictions.

**A.** Intersection between CGH and SNP predictions for genes with more than 4 copies. **B.** Intersection for genes within deletions. **C.** Intersection for genes within deletions for which expression was not detected.
