# Supplementary material for: Network-Guided Analysis of Genes with Altered Somatic Copy Number and Gene Expression Reveals Pathways Commonly Perturbed in Metastatic Melanoma
Source: PLoS One. 2011 Apr 8;6(4):e18369. doi: 10.1371/journal.pone.0018369 (PMC3072964; doi:10.1371/journal.pone.0018369)
Supplement: Figure S8 — Copy number prediction from CGH and SNP arrays, LOH prediction from SNP arrays. (PDF) [file pone.0018369.s008.pdf]

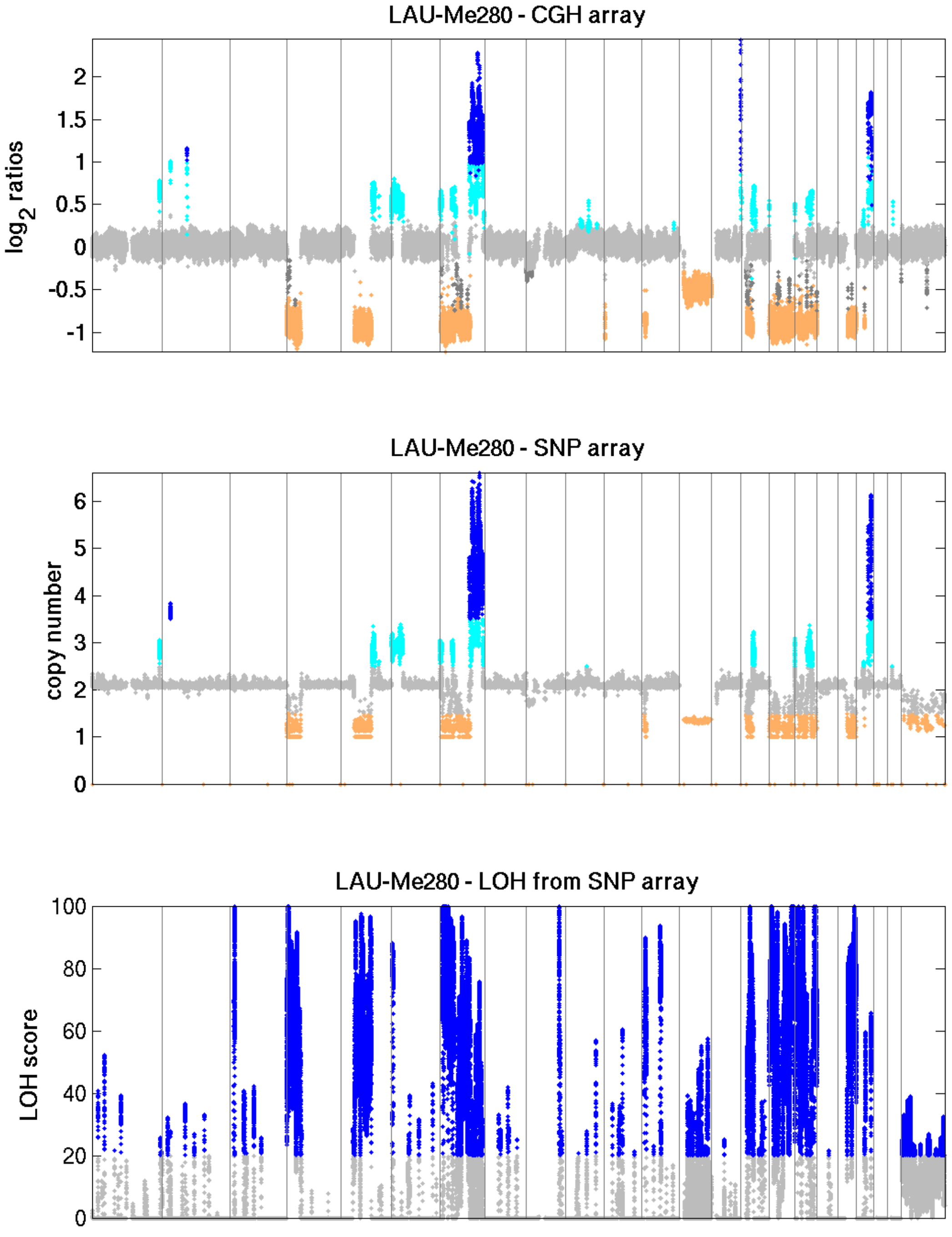

LAU-Me280 - CGH array
log2 ratios
2
1.5
1
0.5
0
-0.5
-1
LAU-Me280 - SNP array
copy number
6
5
4
3
2
1
0
LAU-Me280 - LOH from SNP array
LOH score
100
80
60
40
20
0

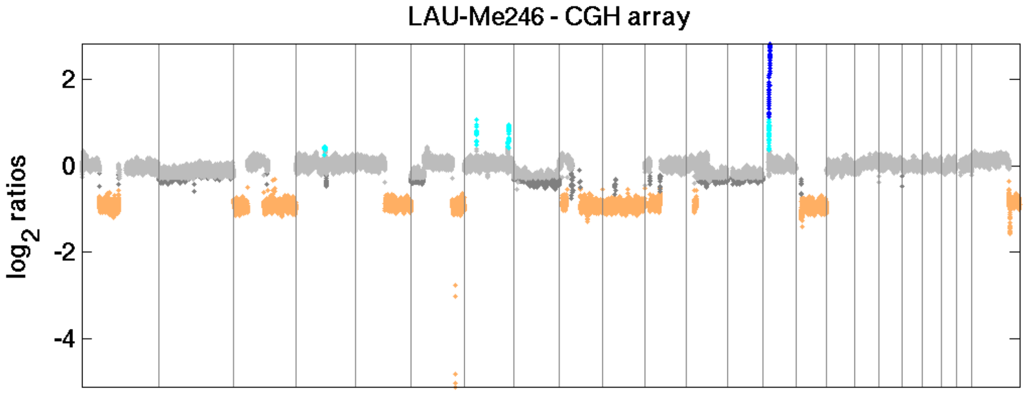

LAU-Me246 - CGH array
log2 ratios
2
0
-2
-4

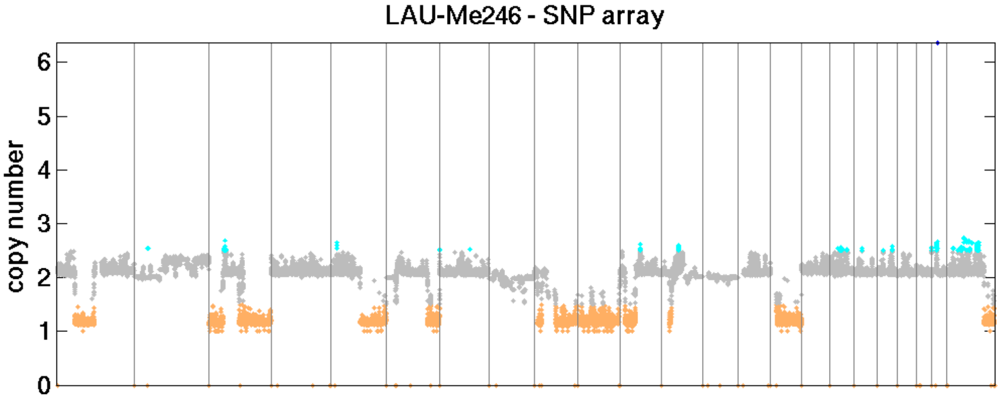

LAU-Me246 - SNP array
copy number
6
5
4
3
2
1
0

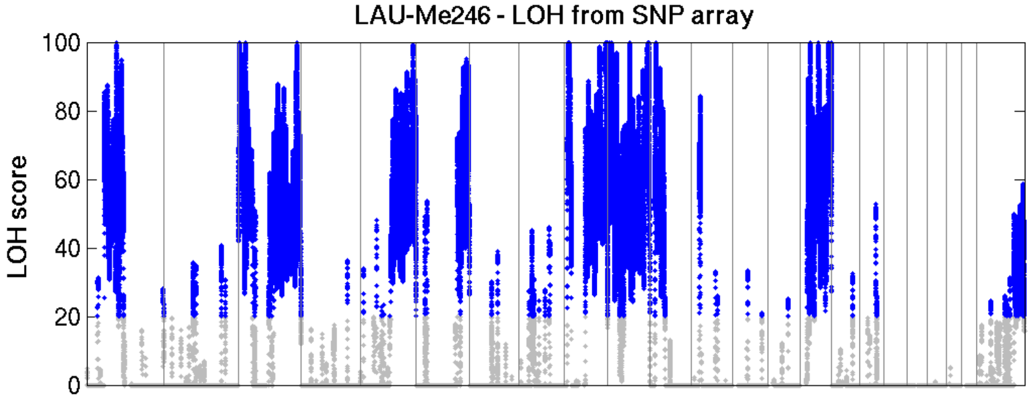

LAU-Me246 - LOH from SNP array
LOH score
100
80
60
40
20
0

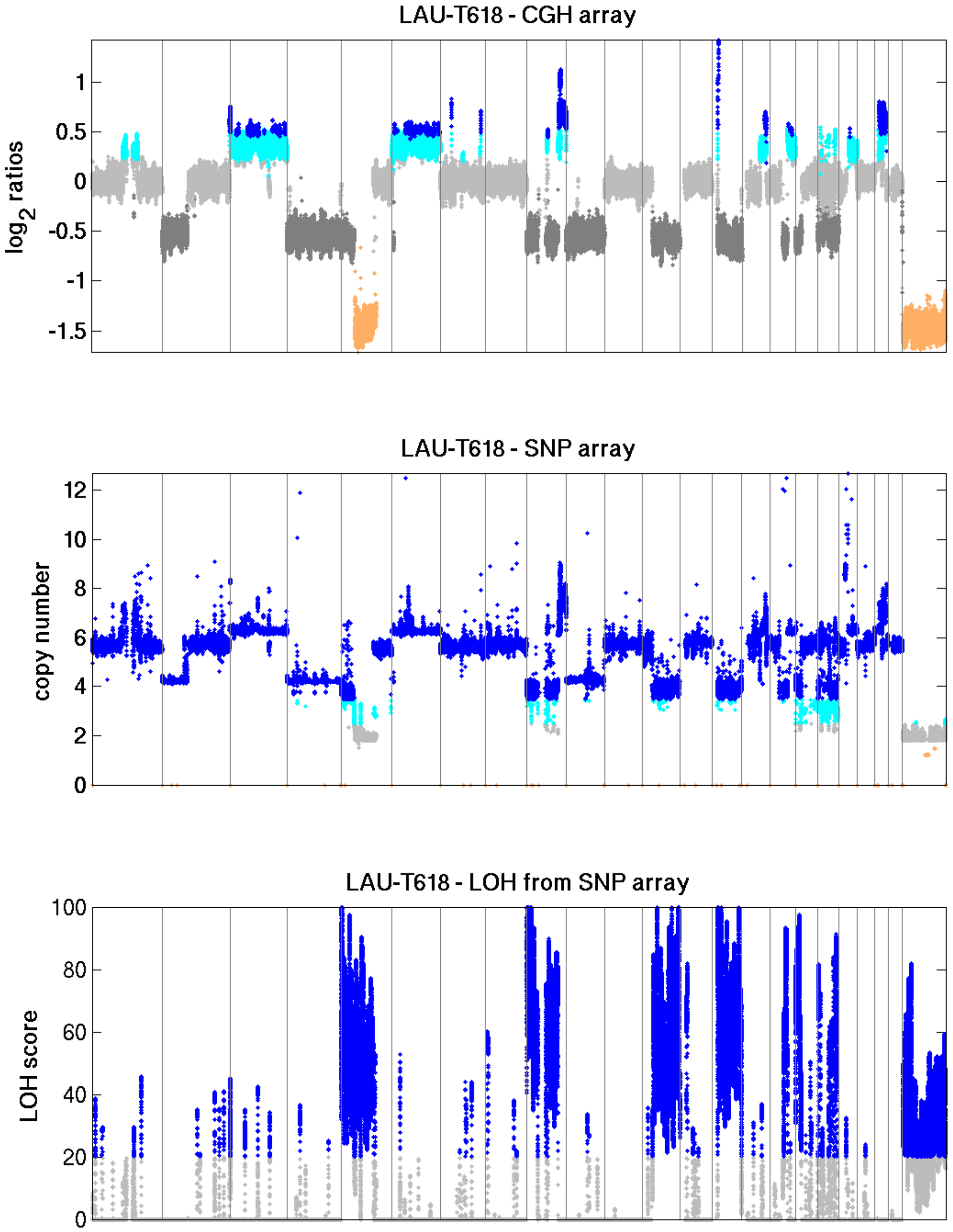
LAU-T618 - CGH array
log2 ratios
1
0.5
0
-0.5
-1
-1.5
LAU-T618 - SNP array
copy number
12
10
8
6
4
2
0
LAU-T618 - LOH from SNP array
LOH score
100
80
60
40
20
0

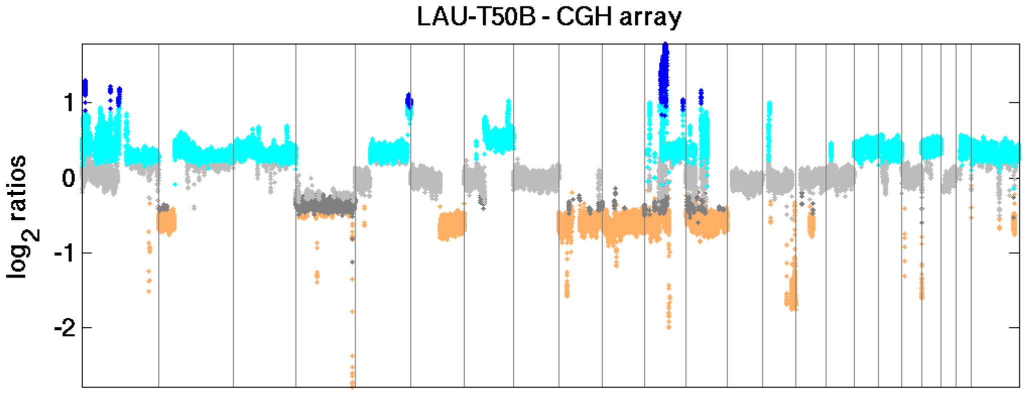
LAU-T50B - CGH array
log2 ratios
1
0
-1
-2

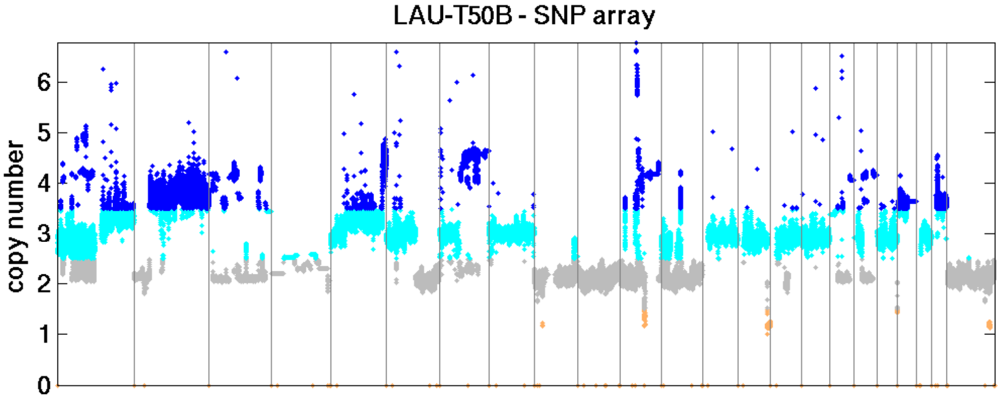
LAU-T50B - SNP array
copy number
6
5
4
3
2
1
0

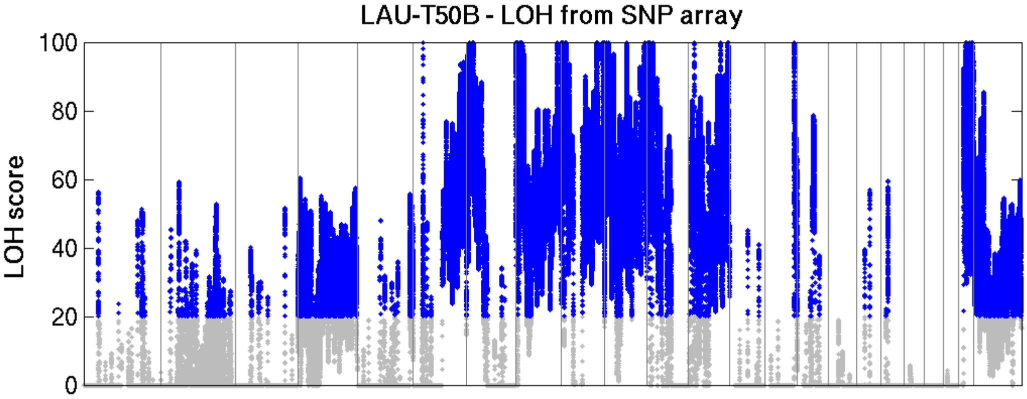
LAU-T50B - LOH from SNP array
LOH score
100
80
60
40
20
0

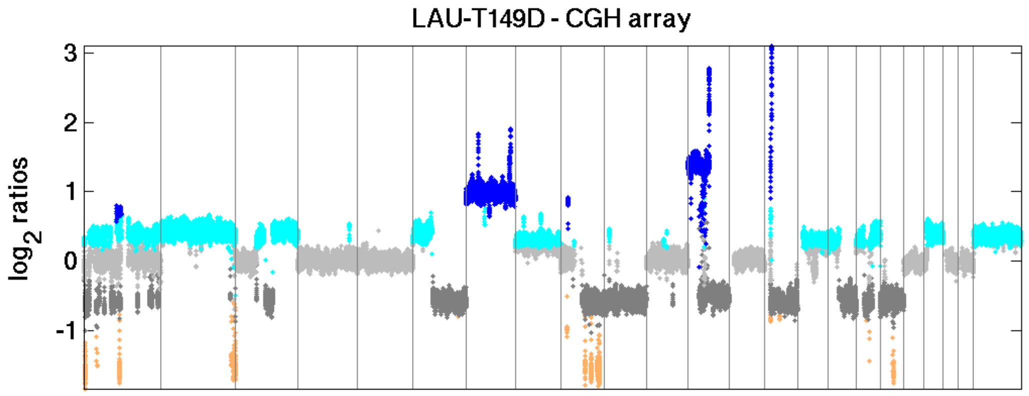
LAU-T149D - CGH array
log2 ratios
3
2
1
0
-1

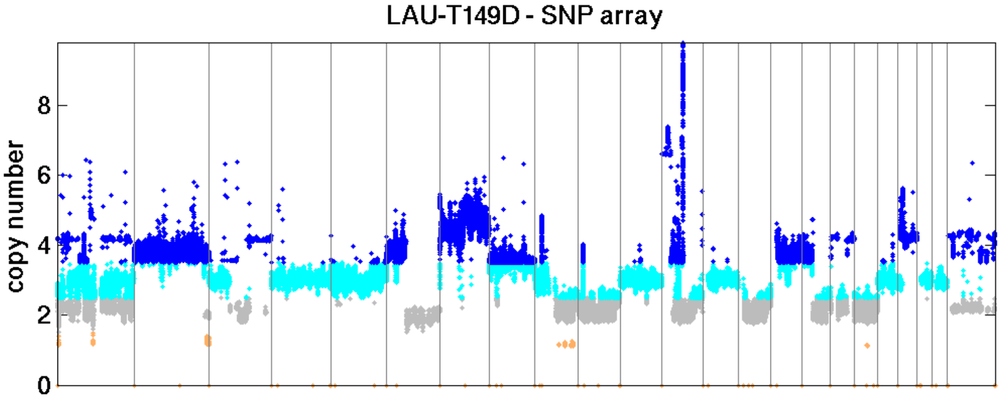
LAU-T149D - SNP array
copy number
8
6
4
2
0

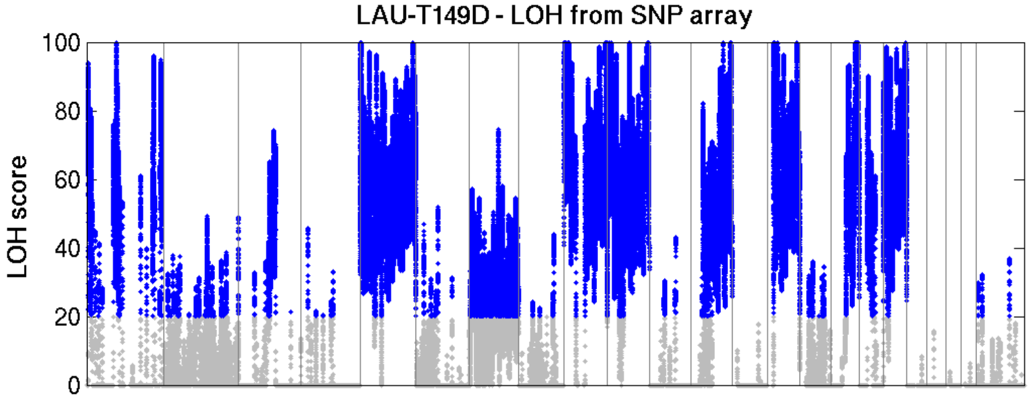
LAU-T149D - LOH from SNP array
LOH score
100
80
60
40
20
0

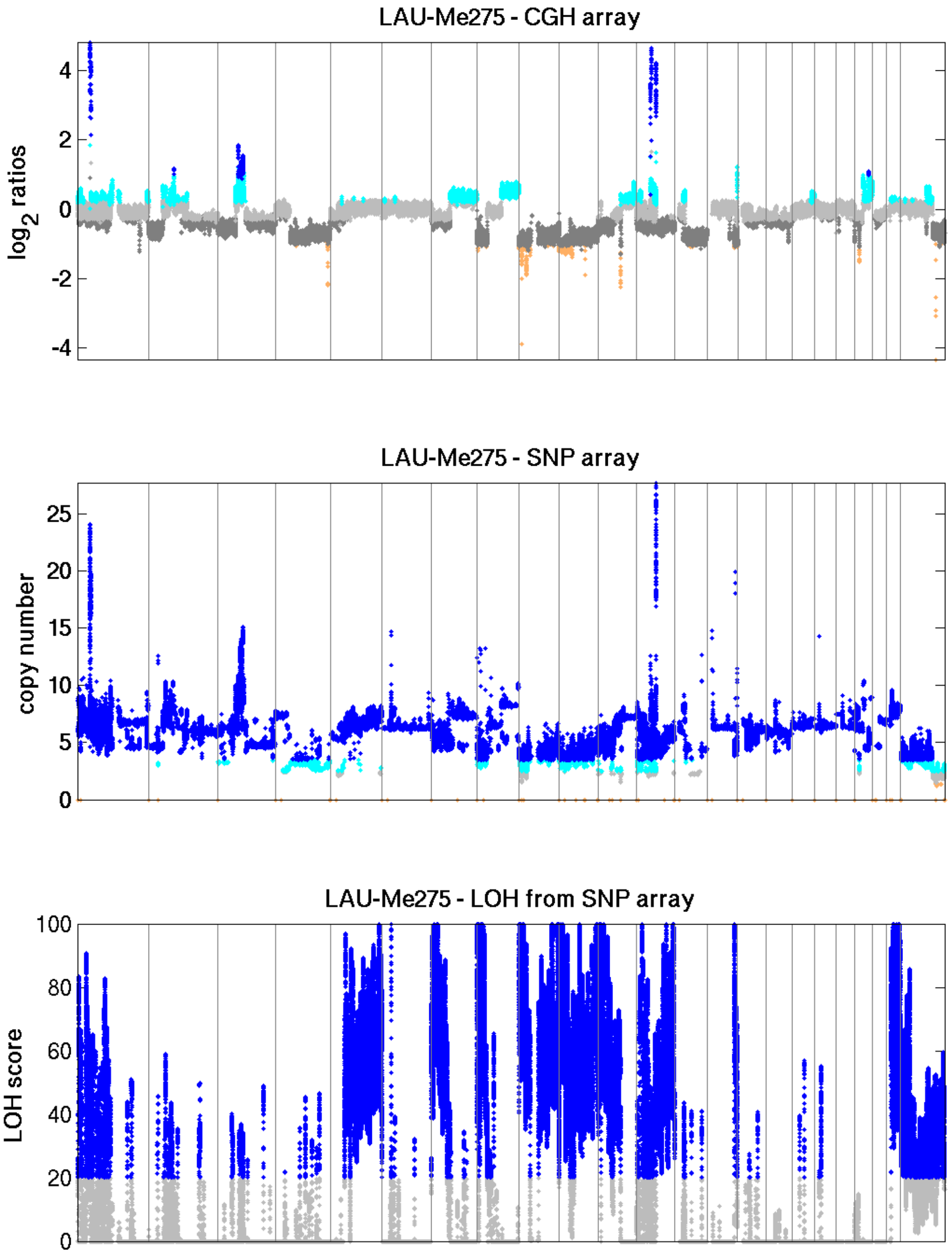

LAU-Me275 - CGH array
log2 ratios
4
2
0
-2
-4
LAU-Me275 - SNP array
copy number
25
20
15
10
5
0
LAU-Me275 - LOH from SNP array
LOH score
100
80
60
40
20
0

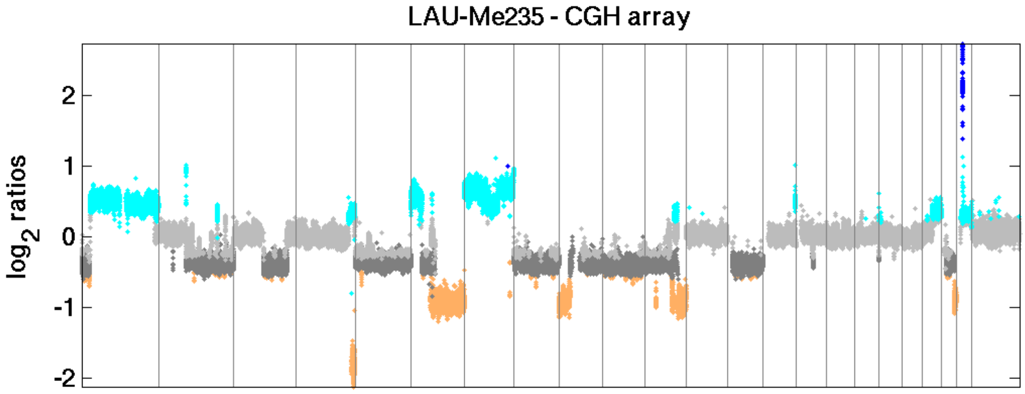
LAU-Me235 - CGH array
log2 ratios
2
1
0
-1
-2

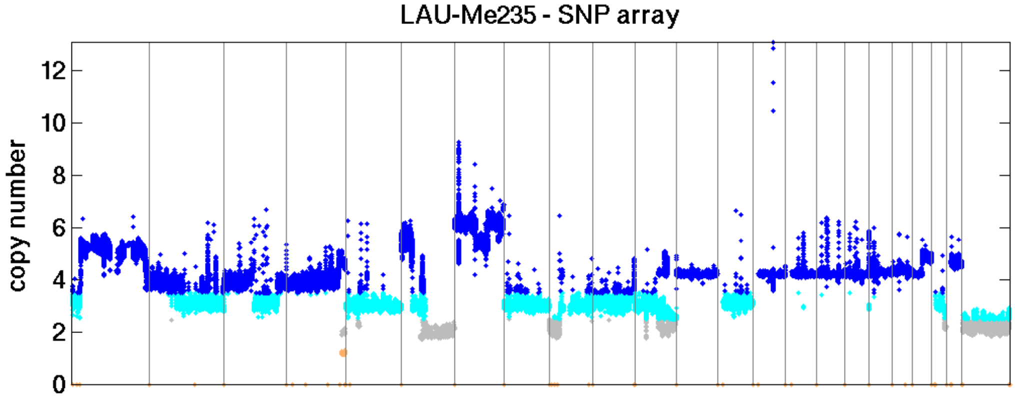
LAU-Me235 - SNP array
copy number
12
10
8
6
4
2
0

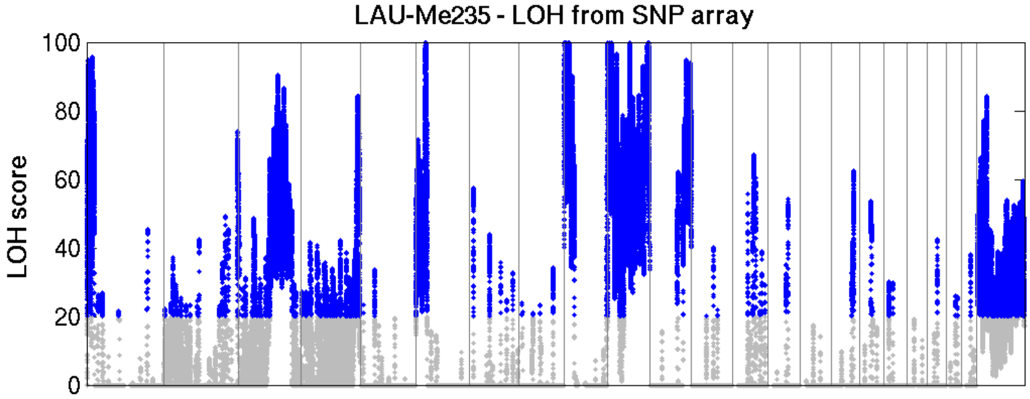
LAU-Me235 - LOH from SNP array
LOH score
100
80
60
40
20
0
