# Supplementary material for: Network-Guided Analysis of Genes with Altered Somatic Copy Number and Gene Expression Reveals Pathways Commonly Perturbed in Metastatic Melanoma
Source: PLoS One. 2011 Apr 8;6(4):e18369. doi: 10.1371/journal.pone.0018369 (PMC3072964; doi:10.1371/journal.pone.0018369)
Supplement: Figure S9 — Boxplots of SILAC heavy/light normalized log2 ratios. In all experiments, LAU-Me275 was labeled with the heavy isotope; the unlabeled sample is indicated in the boxplot label on the X axis. ‘NHM’ refers to the pool of normal melanocytes and ‘self-self’ to a control experiment using only LAU-Me275 to check for any bias due to the label/no label culture conditions (no significant bias was detected). (DOC) [file pone.0018369.s009.doc]

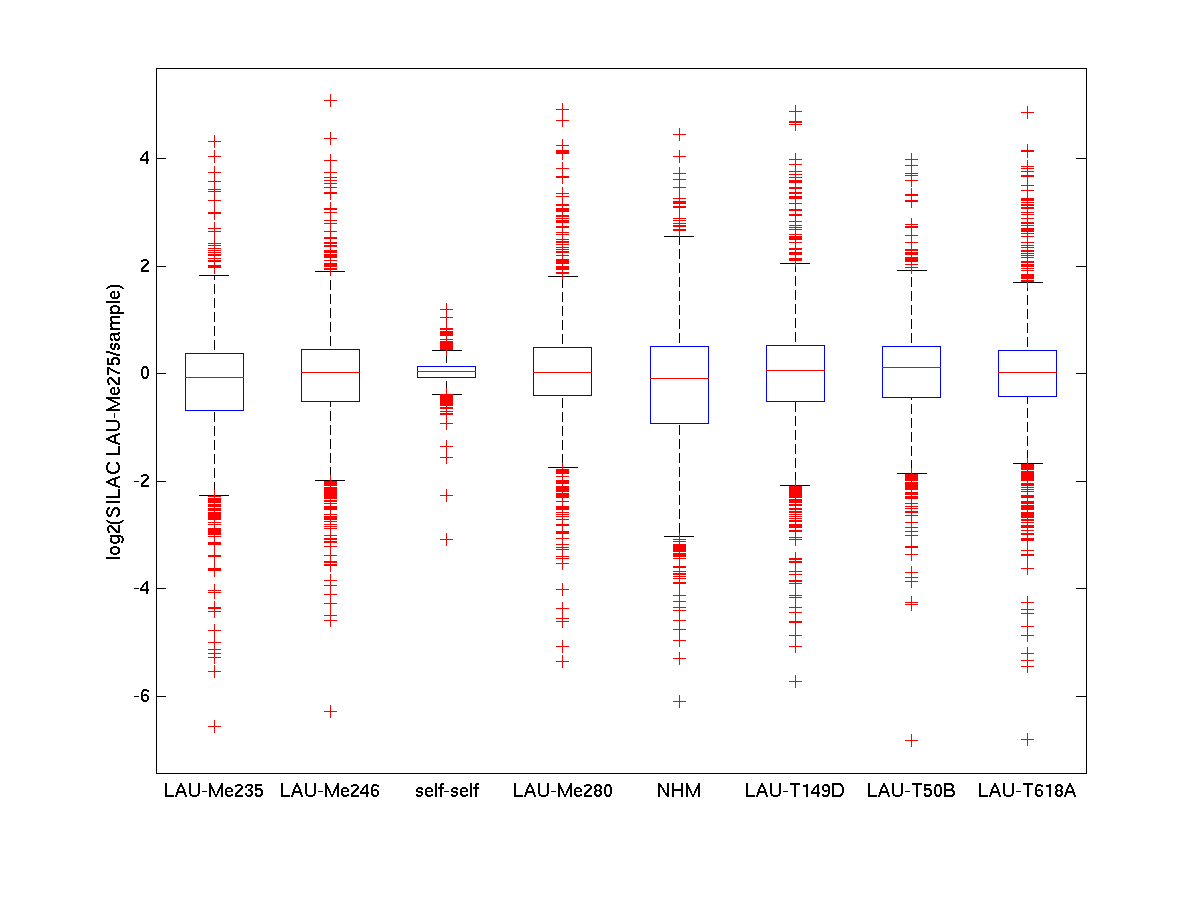


**Figure S9 Boxplots of SILAC heavy/light normalized log2 ratios.**

In all experiments, LAU-Me275 was labeled with the heavy isotope; the unlabeled sample is indicated in the boxplot label on the X axis. ‘NHM’ refers to the pool of normal melanocytes and ‘self-self’ to a control experiment using only LAU-Me275 to check for any bias due to the label/no label culture conditions (no significant bias was detected).
