# Supplementary figures and images for: Network-Guided Analysis of Genes with Altered Somatic Copy Number and Gene Expression Reveals Pathways Commonly Perturbed in Metastatic Melanoma
Source: PLoS One. 2011 Apr 8;6(4):e18369. doi: 10.1371/journal.pone.0018369 (PMC3072964; doi:10.1371/journal.pone.0018369)

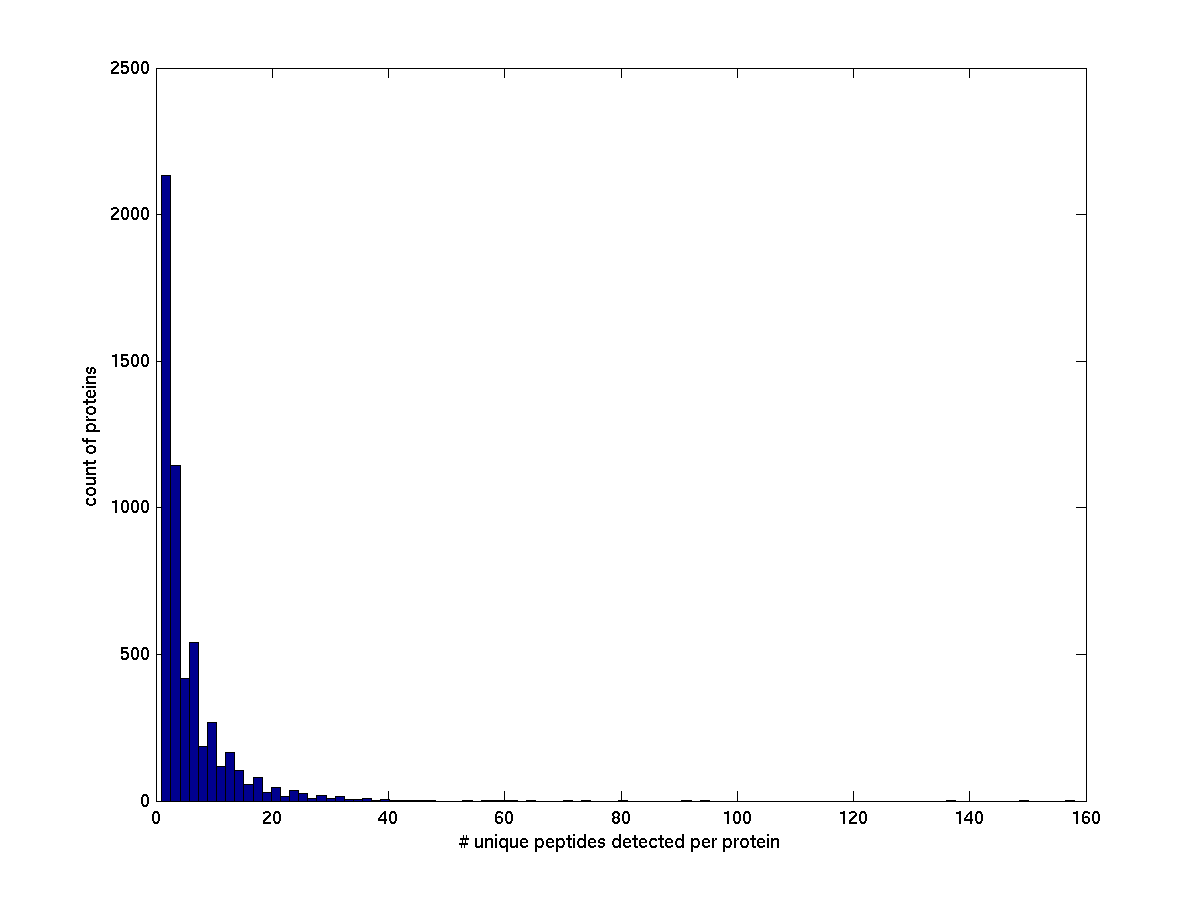


**Figure S10 Histogram of unique peptides identified per protein in the SILAC data.**

Supplement: Figure S10 — Histogram of unique peptides identified per protein in the SILAC data. (DOC) [file pone.0018369.s010.doc]
