# Supplementary material for: Network-Guided Analysis of Genes with Altered Somatic Copy Number and Gene Expression Reveals Pathways Commonly Perturbed in Metastatic Melanoma
Source: PLoS One. 2011 Apr 8;6(4):e18369. doi: 10.1371/journal.pone.0018369 (PMC3072964; doi:10.1371/journal.pone.0018369)
Supplement: Figure S11 — Correlation between mRNA expression and protein levels. Both SILAC and RNA seq log2 ratios are expressed for LAU-Me275 with respect to the sample indicated in each plot title. Spearman Rho correlation coefficient is also indicated in the title. In all experiments, the correlation is significantly positive (p<0.001). (DOC) [file pone.0018369.s011.doc]

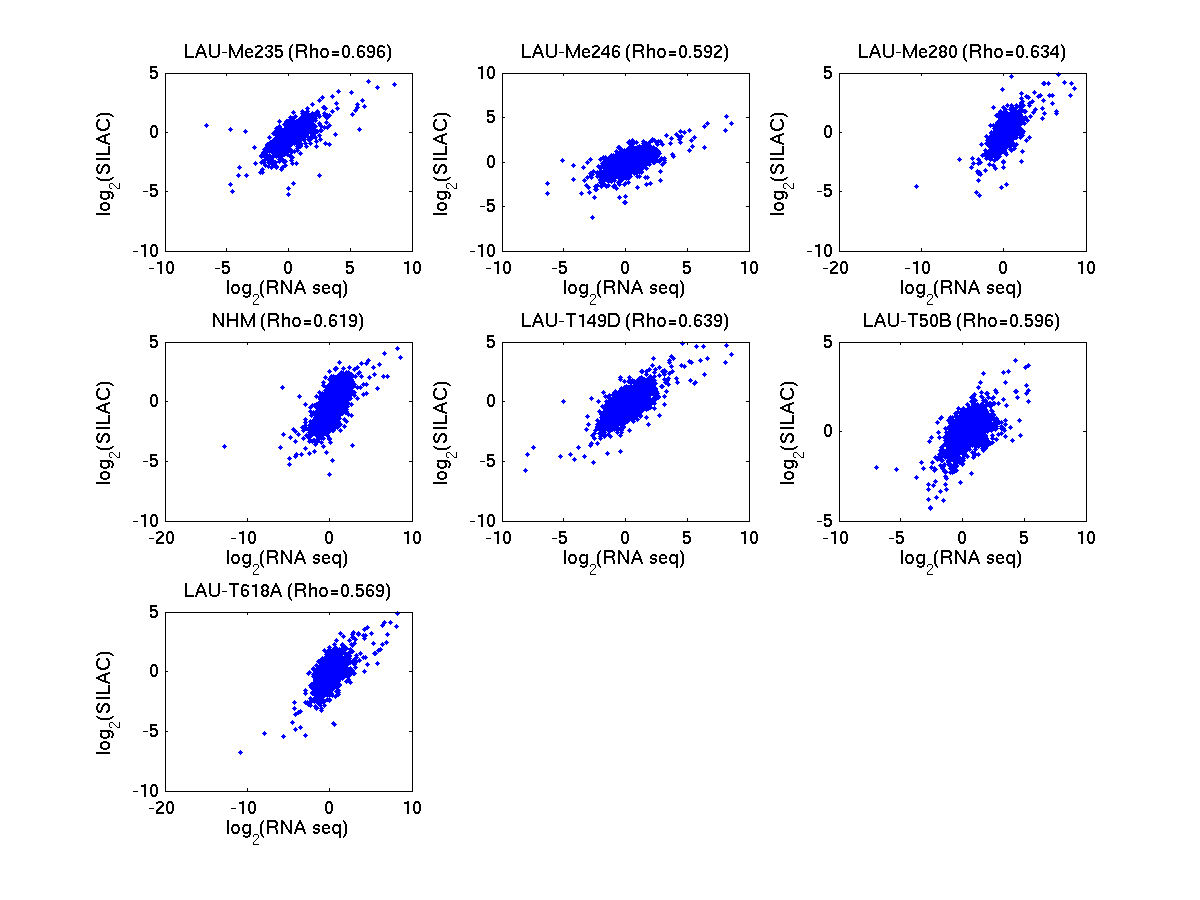


**Figure S11 Correlation between mRNA expression and protein levels.**

Both SILAC and RNA seq log2 ratios are expressed for LAU-Me275 with respect to the sample indicated in each plot title. Spearman Rho correlation coefficient is also indicated in the title. In all experiments, the correlation is significantly positive (p<0.001).
