# Supplementary material for: Network-Guided Analysis of Genes with Altered Somatic Copy Number and Gene Expression Reveals Pathways Commonly Perturbed in Metastatic Melanoma
Source: PLoS One. 2011 Apr 8;6(4):e18369. doi: 10.1371/journal.pone.0018369 (PMC3072964; doi:10.1371/journal.pone.0018369)
Supplement: Table S1 — Count of genes affected by SCNA. (DOC) [file pone.0018369.s012.doc]

|  | **SNP arrays** | | | | | | | |
| --- | --- | --- | --- | --- | --- | --- | --- | --- |
|  | **LAU-Me280** | **LAU-Me246** | **LAU-T618A** | **LAU-T50B** | **LAU-T149D** | **LAU-Me275** | **LAU-Me235** | **unique gene count** |
| **Focal Amplification** | 213 | 0 | 978 | 438 | 894 | 1853 | 161 | 4055 |
| **Focal Amplification with 2xOE** | 85 | 0 | 227 | 106 | 202 | 502 | 25 | 1089 |
| **Arm-level Amplification** | 0 | 0 | 16584 | 1033 | 3477 | 16398 | 10384 | 19496 |
| **Arm-level Amplification with 2xOE** | 0 | 0 | 2988 | 263 | 915 | 3566 | 1778 | 6007 |
| **Deletion** | 2294 | 3157 | 2 | 113 | 70 | 2 | 39 | 5544 |
| **Deletion w/o expression in melanoma but some in melanocytes** | 167 | 157 | 0 | 6 | 3 | 0 | 4 | 333 |
|  |  |  |  |  |  |  |  |  |
|  | **CGH arrays** | | | | | | | |
|  | **LAU-Me280** | **LAU-Me246** | **LAU-T618A** | **LAU-T50B** | **LAU-T149D** | **LAU-Me275** | **LAU-Me235** | **unique gene count** |
| **Focal Amplification** | 0 | 0 | 0 | 26 | 379 | 0 | 4 | 409 |
| **Focal Amplification with 2xOE** | 0 | 0 | 0 | 6 | 129 | 0 | 1 | 136 |
| **Arm-level Amplification** | 222 | 0 | 549 | 99 | 998 | 42 | 0 | 1884 |
| **Arm-level Amplification with 2xOE** | 92 | 0 | 148 | 32 | 398 | 29 | 0 | 689 |
| **Deletion** | 3668 | 4281 | 986 | 3656 | 108 | 122 | 1059 | 10711 |
| **Deletion w/o expression in melanoma but some in melanocytes** | 240 | 208 | 18 | 185 | 5 | 5 | 63 | 634 |

Table S1. Count of genes affected by SCNA.
