# Supplementary material for: Network-Guided Analysis of Genes with Altered Somatic Copy Number and Gene Expression Reveals Pathways Commonly Perturbed in Metastatic Melanoma
Source: PLoS One. 2011 Apr 8;6(4):e18369. doi: 10.1371/journal.pone.0018369 (PMC3072964; doi:10.1371/journal.pone.0018369)
Supplement: Methods S1 — (DOC) [file pone.0018369.s017.doc]

# Supplemental Information

# Network-guided analysis of genes with altered somatic copy number and gene expression reveals pathways commonly perturbed in metastatic melanoma

Armand Valsesia^1,2,3^, Donata Rimoldi^1^, Danielle Martinet^4^, Mark Ibberson^2^, Paola Benaglio^3^, Manfredo Quadroni^5^, Patrice Waridel^5^, Muriel Gaillard^4^, Mireille Pidoux^4^, Blandine Rapin^4^, Carlo Rivolta^3^, Ioannis Xenarios^2^, Andrew J.G. Simpson^6^, Stylianos E. Antonarakis^7^, Jacques S. Beckmann^3,4^, C. Victor Jongeneel^1,2,8^, Christian Iseli^1,2^*, and Brian J. Stevenson^1,2^*

## Supplementary Methods

### CNV analysis from CGH arrays

Hybridization signals were extracted using the Feature Extraction software (v.9.5.3.1) and normalized using three independent methods: 1) the local weighted polynomial regression (Loess [1]), widely used for the analysis of diploid genomes; 2) the PopLowess method proposed by Staaf et al [2], where normalization is applied to population of probes that have been clustered in a deletion, copy neutral or duplication bin; and 3) the more elaborated framework from Chen et al [3], which combines several approaches to calibrate channels from all arrays and to centralize the copy number ratios.

After normalization, we segmented the log2 ratios using Circular Binary Segmentation [4,5] (with parameters undo.splits=”sdundo”, undo.SD=2, nperm=10000 and alpha=0.01) and attributed a discrete copy number to segments using three independent methods:

1) Scoring-based approach.

We computed a score S defined as :

$$S=\frac{r-median(R)}{mad(R)}$$

where R is the log2 ratio for a chromosome, r the median log2 ratio for a CBS segment, and mad the median absolute deviation, a robust estimator of dispersion around the median. This score reflects how significant a segment is compared to the chromosome baseline, segments with S <-4 were classified as CN=0; S <-2 as CN=1; S >2 as CN=3 and S >4 as CN=4.

2) The MergeLevels method from Willenbrock and Fridlyand [6]. This procedure is used to effectively remerge similar segments and to produces a new segment level that is used for classification into deletion and duplication events. This procedure has been used in several CNV detection frameworks [7,8].

3) Classification based on Gaussian Mixture Model (GMM).

GMM fit Gaussian Model on the log2 ratios from CBS segments to identify Gaussian components in the distribution (Supplemental Fig. S3). Several models (with different numbers of components) are fitted using an expectation maximization algorithm [9], only the model that minimizes the Bayesian Information Criteria [10] is kept for subsequent segment clustering. The cluster with the median log2 ratio the closest to zero is assumed to reflect copy neutral events (CN=2). The right-hand side cluster (with positive ratios) is assumed to reflect duplication events (CN=3), any additional clusters with higher ratios are classified as amplification events (CN≥4).

An interesting property of the GMM is its ability to detect copy neutral events due to cell heterogeneity. We initially thought the GMM clusters with negative ratios would only reflect deletion events. In fact, karyotype analysis revealed that the component left of the diploid state was reflecting mostly diploid events and few deletions. For example, in LAU-Me275, Chr4q and Chr10q both had mean log2 ratios close to -0.8 (see Fig. 2) and were diploid in 12 and 13 karyotype spreads, respectively (out of 19 spreads), duplicated in 6 and 5 spreads and deleted in one. By contrast, in LAU-Me280, Chr13q had a mean log2 ratio close to -0.5, was deleted in 10 out of 15 karyotype spreads and its corresponding GMM component was not the adjacent neighbor of the diploid component. This demonstrates that setting thresholds on log ratios is not appropriate and that the ratios should be modeled within a sample and not across samples. In this case, statistical decomposition of the data (e.g. using GMM) is helpful to distinguish between genuine copy number events and loci that are mostly diploid in a cell population but can undergo sporadic copy number events. Based on this observation, we assigned the cluster left of the diploid component as copy neutral, and any cluster with a more negative ratio was classified as a deletion event (CN<2).

### Transcriptome analysis

**cDNA preparation**

mRNA isolation and cDNA preparation were performed following the protocol used by Bainbridge et al [11], with some modifications. Specifically, mRNA was purified from 300-500 µg of total RNA from each sample using the µMACS mRNA Isolation Kit (Miltenyi Biotec, Bergisch Gladbach, Germany), inclusive of the optional DNase I treatment (2 U of DNase I, RNase free, Roche, Mannheim, Germany) during the purification. The quality and amount of purified mRNA were determined using a Bioanalyzer 2100 (RNA Nano assay, Agilent Technologies, Basel, Switzerland). cDNA was prepared from mRNA (2-5µg), using the SuperScript Double-Stranded cDNA Synthesis Kit (Invitrogen Life Technologies, Carlsbad, CA), and quantified with Quant-iT PicoGreen (Invitrogen). The resulting 3-5 µg of cDNA were used for Roche 454 library preparation, according to the manufactures’ procedures. RNA preparations from two normal melanocytecell lines were pooled together and purified as a single sample.

**Sequence analysis**

Sequences from the Refseq, GeneBank RNA and ESTs databases, as well as 454 reads obtained from the melanoma and melanocyte cell line cDNAs were aligned to the the GRCh37 assembly of the human genome using SIBsim4, a modified version of sim4 [12,13]. For sequences that matched to multiple locations, only the best alignment was kept. Finally Refseq mRNAs were used to annotate these unique transcripts and to compute a sequence tag count per transcript and per sample analyzed.

### Protein network-guided analysis

We mapped SCNA onto our non-redundant human protein interaction network and identified connected components using the RBGL package [14]. Putative functional clusters within these networks were calculated using the walk trap community algorithm from the igraph package [15,16]. For community detection a random walk path length of 3 was used and functional clusters were extracted where the modularity was maximal. Clusters with less than 5 nodes were filtered out and not used for further analysis. To test the significance of the clustering, a permutation test was performed by re-calculating the clustering of 1000 random networks generated from the original subnetwork. The resulting random networks had randomized edges but the same degree distribution as the original subnetwork.

### SILAC analysis

Cells were grown in RPMI (Cell Culture Technologies, Gravesano, Switzerland) with 10% (v/v) dialyzed FBS (Invitrogen). Isotope-labeled amino acids (Lysine ^13^C_6_, Arginine ^13^C_6_^15^N_4_, Cambridge Isotope Laboratories, Andover, MA) were added in the “heavy” medium at their normal RPMI concentrations, while proline was supplied at double concentration in all media. The cell line LAU-Me275 was heavy labeled by culturing in “heavy medium” for 18-20 days to allow for at least five cell divisions and used as reference for all comparisons. The other lines were grown in light medium (same as “heavy medium” but containing normal lysine and arginine) for 3-6 weeks to achieve comparable conditions, except for normal melanocytes, which were cultured as described in the Methods’ section. Cells were harvested using Accutase (Thermo) and rinsed three times with PBS before lysis.

Cell pellets were lysed by boiling and sonication in 4% SDS, 100mM Tris/HCl pH 7.5, 100 mM DTT. After centrifugation and protein concentration measurements, equimolar extracts from light/heavy labeled cells were combined and digested as described [17]. The obtained peptide mixtures (200ug total material) were desalted on SepPak C18 cartridges (Waters Corp., Milford, MA), dried, redissolved in 4M Urea with 0.1% Ampholytes pH 3-10 (GE Healthcare) and fractionated by off-gel focusing as described [18]. The 24 fractions obtained were desalted on a microC18 96-well plate (Waters Corp.,Milford, MA), dried and resuspended in 0.1% formic acid, 3% (v/v) acetonitrile for LC-MS analysis.

Samples were analyzed on a hybrid linear trap LTQ-Orbitrap XL mass spectrometer (Thermo Fisher, Bremen, Germany) interfaced *via* a TriVersa Nanomate (Advion Biosciences, Norwich, UK) to a Agilent 1100 nano HPLC system (Agilent Technologies, Waldbronn, Germany).

Peptides were separated on a reversed-phase nanocolumn ZORBAX 300SB C18 column (75 μm ID x 15 cm, 3.5 μm, Agilent) with a gradient from 5 to 85 % acetonitrile in 0.1% formic acid (total time: 120 min). In data-dependent acquisition controlled by Xcalibur 2.0.7 software (Thermo Fisher), the ten most intense multiply charged precursor ions detected in the full MS survey performed in the Orbitrap were selected for CID fragmentation in the LTQ linear trap and then dynamically excluded from any selection during 120 s.

## Supplementary References

1. Smyth GK, Speed T (2003) Normalization of cDNA microarray data. Methods 31: 265-273.

2. Staaf J, Jonsson G, Ringner M, Vallon-Christersson J (2007) Normalization of array-CGH data: influence of copy number imbalances. BMC Genomics 8: 382.

3. Chen HI, Hsu FH, Jiang Y, Tsai MH, Yang PC, et al. (2008) A probe-density-based analysis method for array CGH data: simulation, normalization and centralization. Bioinformatics 24: 1749-1756.

4. Olshen AB, Venkatraman ES, Lucito R, Wigler M (2004) Circular binary segmentation for the analysis of array-based DNA copy number data. Biostatistics 5: 557-572.

5. Venkatraman ES, Olshen AB (2007) A faster circular binary segmentation algorithm for the analysis of array CGH data. Bioinformatics 23: 657-663.

6. Willenbrock H, Fridlyand J (2005) A comparison study: applying segmentation to array CGH data for downstream analyses. Bioinformatics 21: 4084-4091.

7. Diaz-Uriarte R, Rueda OM (2007) ADaCGH: A parallelized web-based application and R package for the analysis of aCGH data. PLoS One 2: e737.

8. Budinska E, Gelnarova E, Schimek MG (2009) MSMAD: a computationally efficient method for the analysis of noisy array CGH data. Bioinformatics 25: 703-713.

9. Dempster AP, Laird NM, Rubin DB (1977) Maximum Likelihood from Incomplete Data Via Em Algorithm. Journal of the Royal Statistical Society Series B-Methodological 39: 1-38.

10. Schwarz G (1978) Estimating Dimension of a Model. Annals of Statistics 6: 461-464.

11. Bainbridge MN, Warren RL, Hirst M, Romanuik T, Zeng T, et al. (2006) Analysis of the prostate cancer cell line LNCaP transcriptome using a sequencing-by-synthesis approach. BMC Genomics 7: 246.

12. Florea L, Hartzell G, Zhang Z, Rubin GM, Miller W (1998) A computer program for aligning a cDNA sequence with a genomic DNA sequence. Genome Res 8: 967-974.

13. SIBsim4 http://sibsim4sourceforgenet/.

14. Gentleman RC, Carey VJ, Bates DM, Bolstad B, Dettling M, et al. (2004) Bioconductor: open software development for computational biology and bioinformatics. Genome Biol 5: R80.

15. Csárdi G, Nepusz T (2006) The igraph software package for complex network research. InterJournal, Complex Systems 1695.

16. Pons P, Latapy M (2005) Computing communities in large networks using random walks. Computer and Information Sciences - Iscis 2005, Proceedings 3733: 284-293.

17. Wisniewski JR, Zougman A, Nagaraj N, Mann M (2009) Universal sample preparation method for proteome analysis. Nat Methods 6: 359-362.

18. Geiser L, Dayon L, Vaezzadeh AR, Hochstrasser DF (2011) Shotgun proteomics: a relative quantitative approach using Off-Gel electrophoresis and LC-MS/MS. Methods Mol Biol 681: 459-472.
